# Construction and Validation of a Regulatory Network for Pluripotency and Self-Renewal of Mouse Embryonic Stem Cells

**DOI:** 10.1371/journal.pcbi.1003777

**Published:** 2014-08-14

**Authors:** Huilei Xu, Yen-Sin Ang, Ana Sevilla, Ihor R. Lemischka, Avi Ma'ayan

**Affiliations:** 1 Department of Pharmacology and Systems Therapeutics, Icahn School of Medicine at Mount Sinai, New York, New York, United States of America; 2 Department of Developmental and Regenerative Biology, Icahn School of Medicine at Mount Sinai, New York, New York, United States of America; 3 Black Family Stem Cell Institute, Icahn School of Medicine at Mount Sinai, New York, New York, United States of America; The Jackson Laboratory, United States of America

## Abstract

A 30-node signed and directed network responsible for self-renewal and pluripotency of mouse embryonic stem cells (mESCs) was extracted from several ChIP-Seq and knockdown followed by expression prior studies. The underlying regulatory logic among network components was then learned using the initial network topology and single cell gene expression measurements from mESCs cultured in serum/LIF or serum-free 2i/LIF conditions. Comparing the learned network regulatory logic derived from cells cultured in serum/LIF vs. 2i/LIF revealed differential roles for Nanog, Oct4/Pou5f1, Sox2, Esrrb and Tcf3. Overall, gene expression in the serum/LIF condition was more variable than in the 2i/LIF but mostly consistent across the two conditions. Expression levels for most genes in single cells were bimodal across the entire population and this motivated a Boolean modeling approach. *In silico* predictions derived from removal of nodes from the Boolean dynamical model were validated with experimental single and combinatorial RNA interference (RNAi) knockdowns of selected network components. Quantitative post-RNAi expression level measurements of remaining network components showed good agreement with the *in silico* predictions. Computational removal of nodes from the Boolean network model was also used to predict lineage specification outcomes. In summary, data integration, modeling, and targeted experiments were used to improve our understanding of the regulatory topology that controls mESC fate decisions as well as to develop robust directed lineage specification protocols.

## Author Summary

For this study we first constructed a directed and signed network consisting of 15 pluripotency regulators and 15 lineage commitment markers that extensively interact to regulate mouse embryonic stem cells fate decisions from data available in the public domain. Given the connectivity structure of this network, the underlying regulatory logic was learned using single cell gene expression measurements of mESCs cultured in two different conditions. With connectivity and logic learned, the network was then simulated using a dynamic Boolean logic framework. Such simulations enabled prediction of knockdown effects on the overall activity of the network. Such predictions were validated by single and combinatorial RNA interference experiments followed by expression measurements. Finally, lineage specification outcomes upon single and combinatorial gene knockdowns were predicted for all possible knockdown combinations.

## Introduction

mESCs are derived from the inner cell mass of a developing blastocyst and can be propagated indefinitely in culture. Cultured mESCs can contribute to all adult cell populations, including the germ-line. Human ESCs have similar *in-vitro* differentiation potential. It is now established that somatic cells can be reprogrammed into induced pluripotent stem cells (iPSCs) using simple combinations of transcription factors (TFs) [1]–[3] or other methods. Mouse and human iPSCs closely resemble ESCs, potentially removing ethical and tissue rejection barriers to applications in regenerative medicine. In order to harness the full potential of stem cell therapeutics there is a pressing need to further characterize the regulatory topology that controls pluripotency as well as commitment and differentiation to specific lineages. Pluripotency is maintained by a densely interconnected network of auto- and cross-regulatory TFs and other transcription regulators. These TFs and regulators promote the expression of other pluripotency genes and simultaneously suppress the expression of differentiation inducers [4]. To dissect the ESC regulatory topology, genome-wide high-throughput technologies such as cDNA microarrays, RNA-seq, ChIP-seq, immuno-precipitation followed by mass spectrometry (IP-MS) proteomics and phosphoproteomics, inhibitory RNA (RNAi) screens, as well as other emerging technologies have been applied. However, it remains a challenge to integrate multiple datasets, obtained from distinct sources and molecular regulatory layers into a systems level view of ESC regulation. Such data integration is necessary in order to build reliable predictive regulatory models that would provide a global view of the entire system. While static network diagrams can provide snapshot views of the information processing that controls cell fate decisions, it is necessary to develop regulatory models that capture the dynamical behavior of key regulatory components over time.

In recent years, several stem cell-centered dynamical models have been developed from low-throughput functional studies. Most models employed ordinary differential equations (ODEs) and simulate interactions among a small number of well-studied TFs [5]–[7]. For example, a stochastic ODE model that linked Nanog, Oct4/Pou5f1 and Sox2 to an osteoblast differentiation circuit comprised of three additional TFs showed that cells can jump from one state to another if enough noise is added to the system [8]. In general, predictions made from computational dynamical models of embryonic stem cell have not been extensively experimentally validated until very recently. In the past year, few other comprehensive studies that integrated various datasets and constructed larger models of the ESC regulatory circuitry have emerged [9]–[12]. For example, Dowell et al. [11] integrated gene expression, ChIP-seq, protein interactions, RNAi screens and epigenetics markers to build a Bayesian network model of pluripotency genes. Their main focus was comparing human and mouse ESCs and their networks models are static. One of the advantages of their approach is that the network was not determined a priori which allowed the discovery of novel self-renewal and pluripotency components. Dowell et al. also present a database that is similar to our ESCAPE database [13] called StemSite. Lee and Zhou [10] combined ChIP-seq, gene expression and motif finding data to identify pairs of transcription factors that potentially work together within the pluripotency circuitry. They established 27 interactions between 14 factors. Many of the interactions they identified are consistent with our study. In another similar study Dunn et al. [9] implemented a data constrained Boolean model that connected 12 transcription factors through 16 interactions to suggest the minimal possible circuitry required to maintain pluripotency of mESC. In contrast with these studies, our study has primary gene expression data from single cell mESC, we also consider lineage markers and predict lineage propensity after perturbations, and provide extensive experimental validation with double and triple knockdowns followed by gene expression profiling of both pluripotency regulators and lineage markers.

In order to address the need for multi-level data integration and broader experimentally validated dynamical models, we first extracted a signed and directed network from published ChIP-seq and knockdown followed by expression studies. All included 15 pluripotency network nodes and their interactions are supported by ChIP-seq data providing TF/target-gene binding evidence, as well as significant mRNA expression change following depletion or over-expression of a given TF. The ChIP-seq TF/target-gene binding evidence provides the directionality of the edges; whereas the knockdown or over-expression followed by genome-wide expression evidence establishes positive or negative regulatory edge sign. The underlying network regulatory logic was then learned using single-cell gene expression data collected with a microfluidic device. The learned model was validated by comparing predictions from *in silico* single or combinatorial node knockdowns to experimental single or combinatorial RNAi knockdowns of selected nodes followed by quantitative PCR expression level measurements of the remaining network components. Finally, lineage specification outcomes of single and combinatorial perturbations were predicted for all possible knockdown combinations.

## Results/Discussion

### Construction of a signed and directed network composed of pluripotency regulators and lineage markers

**Figure 1:**
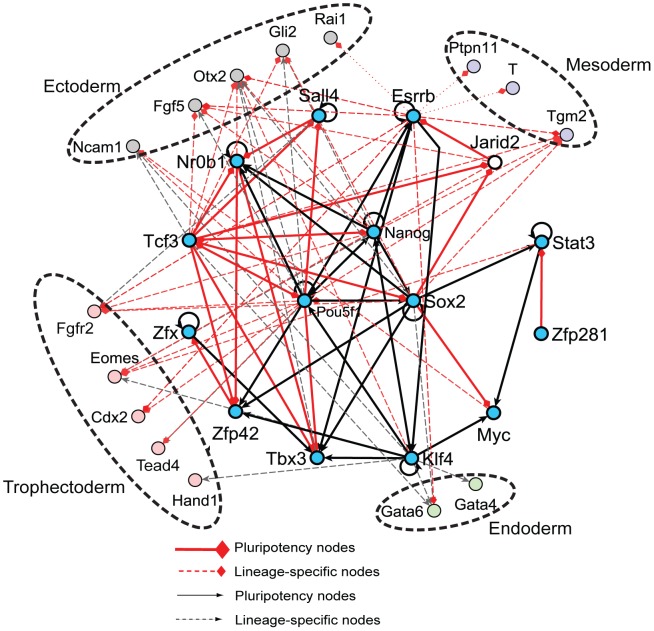
Signed and directed network extracted from ChIP-seq and knockdown or over-expression followed by genome-wide expression. Edges are established where there is evidence for transcription factor binding to the gene proximal region and also change in expression after knockdown or over-expression. The 15 pluripotency nodes are color coded in light blue, and the lineage markers are color coded for the four major early differentiation lineages. Red diamond-heads denote inhibition and black arrowheads denote activation. Dashed lines connect pluripotency regulators to lineage markers and solid arrows connect pluripotency regulators to other pluripotency regulators.

We first extracted a signed and directed pluripotency network of mESCs consisting of 15 transcription regulators and 15 lineage markers from the ESCAPE database (Figure 1) [14]. The ESCAPE database contains TF/target-gene interactions extracted from a collection of ChIP-seq studies applied to mESCs. In addition, the ESCAPE database also contains regulatory causal interactions connecting perturbed TFs to affected target genes based on mRNA expression. Such interactions were extracted from the loss-of-function (LOF) knockdown/knockout of TFs, or gain-of-function (GOF) over-expression followed by global transcriptional profiling measured by microarrays or RNA-seq database tables in ESCAPE. The 15 pluripotency regulators were selected only if both ChIP-seq and LOF/GOF evidence was available; whereas the selection of the 15 lineage markers was determined by expert curation. The network nodes represent 15 of the mostly well-studied pluripotency TFs and 15 of the most established early differentiation lineage markers. The selected factors also have phenotypic evidence that is important for sustaining normal mESC functions based on cell phenotype profiling after knockdown or over-expression. In addition most of the 15 selected pluripotency factors are well studied where some also were shown to play central role in iPSC reprogramming. The 15 lineage markers were selected to be the most established markers with having a relatively even representation for each lineage. The list of studies and the criteria used for inclusion of network nodes are described in Table S1. The initial network created from the high-content studies had few links with conflicting signs. These conflicting signed links were resolved by citing specific publications from the literature (Table S2). The final network contains 30 nodes, 106 links, 10 positive auto-regulatory feedback loops, as well as 26 positive and 13 negative other feedback loops (Figure S1).

### Measuring gene expression in single cells

The 30-node pluripotency network described above can suggest some novel regulatory mechanisms governing mESCs regulation. However, the directed and signed graph representation still lacks crucial regulatory information. Specifically, the transition functions (logic gates) that tie the activity of each node to the activities of its upstream regulators are not specified. In other words, the representation does not provide information on how the combinatorial state of upstream parent nodes determines the expression and activity of a given downstream node. The coding of transcriptional regulation to Boolean logic gates is a mathematical idealization and abstraction of the complex biochemical processes of transcriptional regulations. As such, a Boolean model can capture the essence of the regulatory relationships but may still lose important details. In addition, the network was created from data collected in different laboratories, under different experimental conditions and over different time scales. Moreover, all data were from bulk populations of mESCs and there is evidence for cell-to-cell heterogeneity in mESCs [15], [16]. Hence, some links may be inaccurate, missing, or present in different contexts in individual cells, or in certain subpopulations. We therefore attempted to refine the network topology by learning the transition functions regulatory logic, and as a result enhancing the information about the network links based on gene expression measurements in single mESCs.

**Figure 2:**
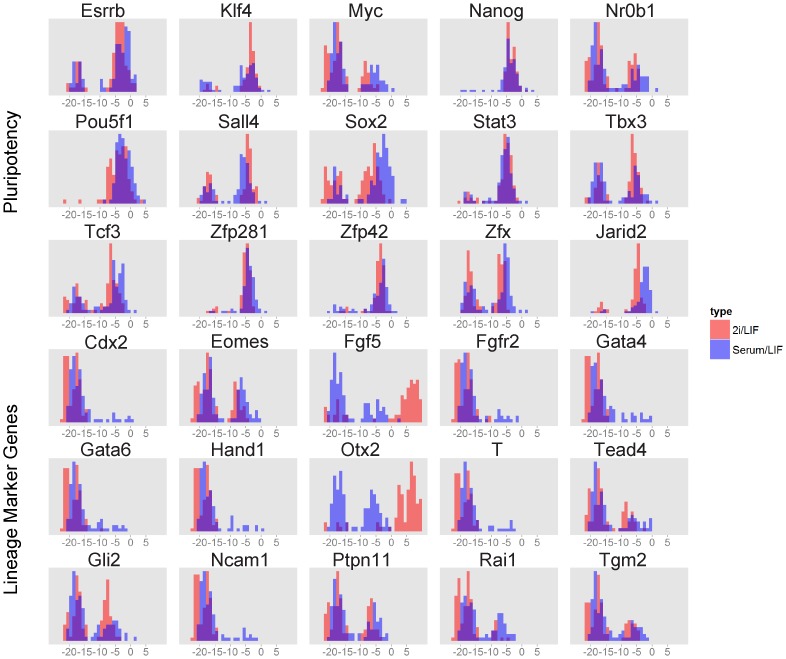
30 mRNA gene expression measurements in 96 single mESCs cell using the Fluidgm single cell microfluidic device. Histograms of gene expression Ct-values from RT-PCR data collected from single mESCs in +serum/LIF (serum/LIF, blue) and -serum/2i/LIF (2i/LIF, red). Ct-values were normalized using the housekeeping gene *Gapdh*. x-axis represents -ΔCt values and y-axis represents the percentage of total cells. Alpha blending is used to show the results from the other condition for clearer visual comparison.

**Figure 3:**
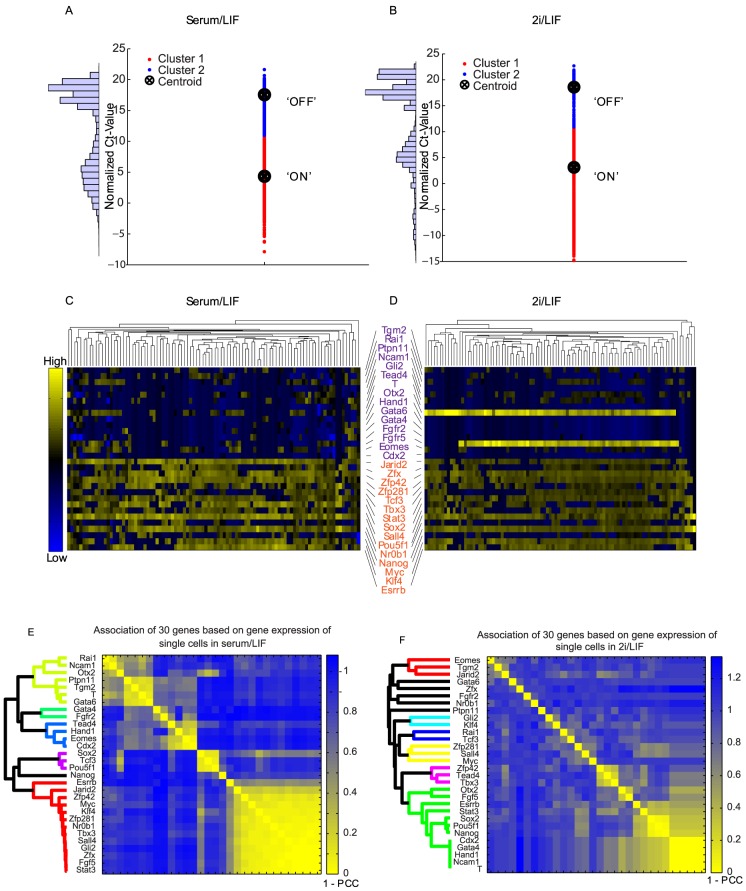
(A-B) Plots of normalized Ct-values partitioned into two clusters. (C–D) Hierarchical clustering of gene expression in single mESCs using normalized Ct-values from RT-PCR data measuring levels of 15 pluripotency genes (orange) and 15 lineage-specific genes (purple) in 96 cells cultured in serum/LIF (C) and in 2i/LIF (D). The x-axis represents individual cells and the y-axis represents genes. Yellow represents ‘high’ and blue represents ‘low’ levels of gene expression. (E–F) Association of 30 genes based on gene expression in single mESCs cultured in serum/LIF or 2i/LIF. Numeric values in the color bars represent the distance score calculated as 1 – Pearson-Correlation Coefficient (PCC). Average-linkage, Euclidean-distance-based hierarchical clustering was performed the gene expression data.

Expression levels were measured using the Fluidigm microfluidic quantitative RT-PCR platform for single cell gene expression profiling [17]. In those experiments the expression of 96 genes was measured in 96 individual cells, including the 30 genes/nodes in the network assembled from ESCAPE (Figures 2, 3, and S2). The remaining 66 genes measured in single cells represent early differentiation markers and controls (Table S3). Measuring 96 individual genes in 96 single cells is a much more direct method to learn the regulatory logic of the pluripotency network. Analysis of data collected from a combined bulk of cells across a set of samples is less direct than single cell data because the regulatory relationships are masked by population averages. Two culture conditions were employed, +serum/LIF (serum/LIF) and –serum/+2i/LIF (2i/LIF). Serum can be a source of variability and with serum-free 2i media, two pharmacological inhibitors targeting the kinases MEK and GSK3β are sufficient to maintain pluripotency [18]. Both conditions benefit from the inclusion of LIF. However, it is still not completely clear how such variable conditions alter the connectivity of the core transcriptional network that maintains pluripotency. The various conditions when compared can give us clues about the required core circuitry that is needed to maintain pluripotency [19], as well as the subtle differences that are expected to exist between conditions, particularly at the single cell level.

Heterogeneous gene expression could arise from stochastic fluctuations or from natural global fluctuations in the pluripotent state [20]. As expected, a comparison of gene expression heterogeneity using indicator dispersion indices revealed less heterogeneity in the 2i/LIF condition as compared to serum/LIF (p<10^−5^, one-tailed paired t-test, Figure S2). This can be seen in the generally narrower distributions for most mRNAs in the 2i/LIF condition (Figure 2). Our results show that Nanog and Zfp42/Rex1 expression is more homogeneous in the 2i/LIF condition compared with the serum/LIF condition, consistent with a previous study [21]. More globally, with the exception of *Tbx3*, *Fgf5* and *Otx2* (highlighted in bold and blue in Figure S2), gene expression patterns are generally similar in the two conditions in terms of expression distributions (Figure 2). In agreement with previous observations, the 2i/LIF media stimulates the Wnt pathway, known to regulate the expression of *Otx2* and *Fgf5* [22], [23]. Also, certain lineage-specific genes, namely *Fgfr2*, *Gata4*, *T*, *Gata6*, *Hand1* and *Ncam1* display overall lower expression levels in 2i/LIF (Table S5). To address the possibility that these observations may be unique to the type of mESCs we used in our experiments, we compared the *Esrrb* expression level distribution to data from another study [24] (Figure S2 and Text S1). In both cases, *Esrrb* mRNA levels are bi-modal and similarly distributed in single cell populations. In addition, we demonstrated previously that Esrrb protein levels are comparable in the mESCs we used and wild-type cells [25]. Since the distribution of Esrrb mRNA expression is similar in the Esrrb removed (Esrrb_R), CCE and Nanog removed (Nanog_R) mESCs using the same Fluidigm BioMark platform, we believe that Esrrb levels are well controlled in the Esrrb_R cell line and the results can be generalized to wild-type mESCs.

Importantly, single cell expression profiles show bimodal distributions for numerous genes (Figures 2 and S2). Therefore, we reasoned that a Boolean framework for learning the underlying network regulatory logic by assigning values of 1 or 0 to high or low expression states, respectively, was a valid initial approach. Boolean modeling of gene-regulatory networks had first been proposed in the late 1960s [26]. Recently this approach regained popularity with the availability of more detailed systems level experimental data and networks [27]. In order to implement a Boolean representation, continuous Ct-value mRNA expression in single cells were converted to binary values (1 or 0) using a single K-means clustering step for all genes with K = 2 (Figure 3A-B and Figure S2). Delta Ct-values are used instead of the exponential of the delta Ct since this way it is more convenient for binarization. Hierarchical clustering of continuous (Figure 3C–D) and binarized Ct-values (Figure S2) of the 30 network gene-products across the 96 cells show that there are no distinct single cell states but rather a level of variability across most cells. We actually expected to observe distinct subpopulations that represent few cell states. The bimodality in single cells has been observed before for single genes and the theory of having few distinct subpopulations is attractive from a modeling perspective. Yet the results confirm the bimodality of gene expression but not the idea of few distinct subpopulations within mESCs. Such variability may be associated with differentiation priming, perhaps to distinct lineages depending on the exact constellation of ON and OFF regulatory nodes in single cells. Priming toward different lineages is supported by the observation that lineage marker genes of the same lineage cluster together due to their high correlation coefficients of expression across the 96 single cells (Figure 3E–F). For example, the ectoderm marker genes *Rai1, Ncam1* and *Otx2* form a cluster and the trophectoderm marker genes *Tead4, Hand1, Eomes* and *Cdx2* form another cluster.

### Learning the regulatory logic functions inter-connecting pluripotency components from single cell gene expression measurements

**Figure 4:**
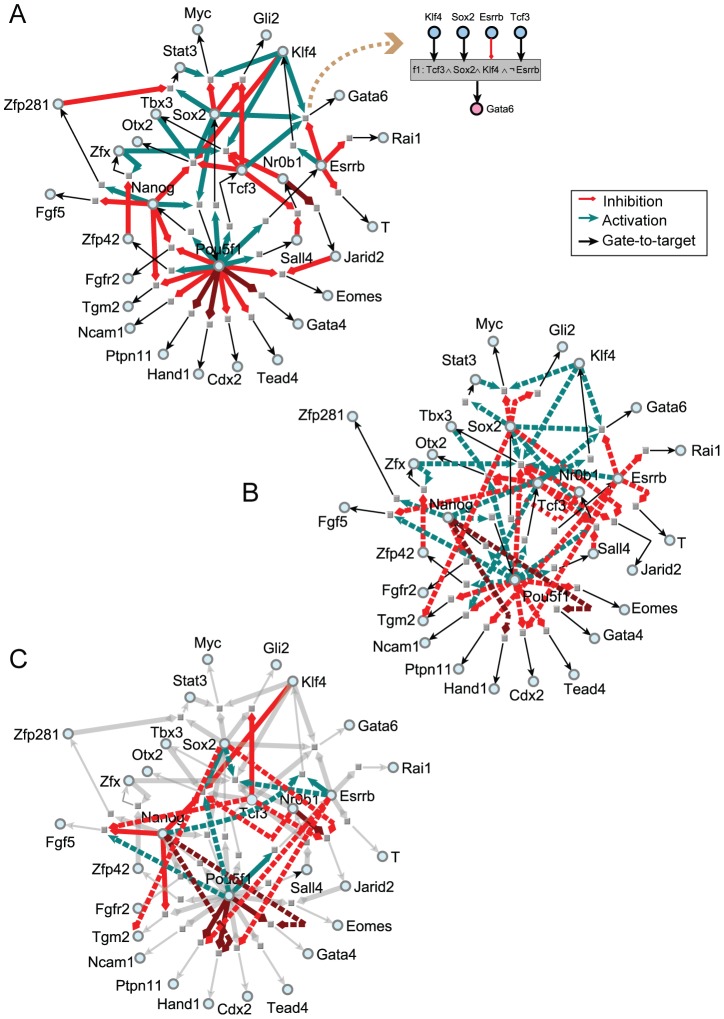
Learned Boolean networks. (A–B) Networks with learned Boolean logic transition functions consisting of 30 genes/proteins. Learning was achieved using the serum/LIF (A) or 2i/LIF (B) single cell data. Light cyan nodes represent genes; gray squares represent learned regulatory logic transition functions. The shadowed inset box exemplifies one such learned transition function upstream of *Gata6*. The sign 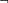 represents ‘NOT’, ∧ represents ‘AND’ and ^∨^ represents ‘OR’ logic operators. Boolean functions for all genes are available in Supplementary Information. Links from upstream parent nodes appearing in more than 90% of equally well-fitted Boolean functions are colored in green (activation) and red (repression). Other links are not shown. Novel links that resulted from the learning process are highlighted in dark red. (C) Overlap between the networks learned using data from mESCs cultured in serum/LIF or 2i/LIF. Links that are shared in the two conditions are in gray. Non-shared links are solid or dashed based on their source (solid for serum/LIF and dashed for 2i/LIF).

To learn the Boolean transition functions upstream of each network node, we applied an exhaustive symbolic search that is limited to the AND, OR and NOT logic operators and composition of these operators with the restriction of allowing each input to feed into only one of gate [28]. Based on the network topology extracted from the ESCAPE database and the single cell data measured with the microfluidic device, we attempted to fit all combinations of the three logic operators for each node given its parental inputs. The learning process used the single cell mRNA levels measured for each gene together with the original topology extracted from the ESCAPE database to derive a truth table for each Boolean transition function composed of AND, OR and NOT operators (Figures 4, S3, and Table S4). We assumed that single cell expression values reflect causal relationships between upstream regulators and downstream targets. Allowing only AND, OR and NOT gates, without nesting them, limits the search space to make the computation sufficiently efficient. However, such simplification may miss important biologically relevant Boolean functions. For example, exclusive OR (XOR) gates likely exist within mammalian cellular gene regulatory networks. In addition, threshold Boolean functions, which are Boolean functions that require at least several but not specific inputs to be active in order to turn on the target output are also missed by our symbolic search. The degeneracy of the possible functions listed in Table S4 suggests that such canalizing functions are likely present in the pluripotency circuits and finding them may reduce the degeneracy and complexity of the ensemble of dynamical Boolean models we obtained. Although there can be many Boolean functions that fit the same experimental observations, we attempted to find those that are most consistent with the direction and sign of edges of the network topology extracted from the ESCAPE database. For some input/output relationships many Boolean functions satisfied the input/output relationships (Table S4). For each gene, if no consistent function emerged with the initial topology, we performed a refinement process. The refinement process starts by testing all possible transition functions using a single regulator, sampling all possible regulators, then pairs and so on, until a defined threshold of input/output agreement was reached. For each gene, if no consistent function emerged, we performed the pruning refinement step by systematically removing all parental input links one-by-one and re-sampling all possible transition functions. This refinement and pruning procedure was executed recursively until a defined threshold of input/output agreement was reached. Specifically, if none of the combinations of plausible parents from the input network fulfilled the criteria, where is the number of single cell expression vectors, the algorithm exhaustively introduces single links from all the 15 pluripotency nodes. If still no single reassigned of a parent can explain the behavior of the downstream target by satisfying the criteria, we attempted all pairs of parents. For only four nodes from the serum/LIF learned network, and two nodes from the 2i/LIF learned network this process was necessary (Table S4). Auto-regulatory interactions are not considered in the dynamic Boolean models to keep the model dynamics simple.

After learning, we obtained a large ensemble of Boolean network models where, in some cases, many distinct transition functions can satisfy the input/output relationships almost equally well. The average number of feedback loops after sampling 100 network models and without considering auto-regulatory loops, was 14.39, including an average of 9.38 positive and 5.01 negative feedback loops (Figure S3). This number of feedback loops is significantly lower than the 39 feedback loops found in the initial network prior to learning the transition functions (p-value <10^−65^, t-test). The initial topology of the network created from the ESCAPE database has many more links than the pruned network, after fitting this network to be in concordance with the single cell data. The pruning and refinement step eliminates links iteratively, from the original topology of the initial network, with the goal of finding transition functions that are consistent with the original topology as well as with the single cell data. We next enumerated all feedback loops present in the sampled Boolean network models and ranked positive and negative loops based on their occurrence (Figure S3). The most common positive feedback loop in randomly selected models is the mutual activation of the *Oct4/Pou5f1* gene by Nanog and the *Nanog* gene by the transcription factor Oct4/Pou5f1; whereas, the most common negative feedback loop is the activation of the gene *Tcf3* by Oct4/Pou5f1 and the inhibition of the *Oct4/Pou5f1* genes by the Tcf3 transcription factor (Figure S3).

While it is established that 2i/LIF can replace serum/LIF to maintain the mESC ground pluripotency state [18], detailed mechanisms responsible for culture-dependant similarities and differences remain elusive. Comparing the networks learned from both conditions, we found that the two networks re generally consistent with 21 out of 30 nodes having exactly the same connectivity. While the original topology of the serum/LIF and 2i/LIF networks is identical, during the pruning and refinement stage, some links can be removed, sign switched or added to obtain transition functions that are consistent with the single cell gene expression data. Therefore, the connectivity of the learned serum/LIF and 2i/LIF networks are slightly different from each other. We observed differences in the predicted regulation of the genes *Klf4*, *Tbx3*, *Jarid2*, *Fgf5*, *Gata4, Hand1*, *Otx2*, *Gli2* and *Ptpn11* (Figure 4). In addition, the regulation by the key TF genes *Oct4/Pou5f1*, *Nanog*, *Sox2*, *Esrrb* and *Tcf3* all showed some level of difference when comparing the two conditions (Figure 4C). For example, Nanog appears to be a positive regulator of *Klf4* and a negative regulator of *Gata4* and *Hand1* only in the 2i/LIF condition, while appearing as a negative regulator of *Fgf5* under the serum/LIF condition. The family of learned Boolean functions confirmed known regulatory interactions and identified new ones. For example, *Oct4/Pou5f1* is positively regulated by Sox2 and Nanog, which is known [29]–[31]. In turn, these links are reinforced by positive regulation of *Sox2* and *Nanog* by Oct4/Pou5f1. Furthermore, Esrrb was singled out as an activator of *Klf4*; whereas before learning, Nanog was a second potential *Klf4* regulator. Direct activation of *Klf4* by Esrrb may explain the ability of Esrrb to replace Klf4 for iPSC reprogramming [32]. Similarly, the repressive regulation of *Nr0b1* (*Dax1*) by Tcf3 and Sall4 was highlighted after refining the network topology with single cell data. Based on out-degree centrality (direct targets per TF), Oct4/Pou5f1 emerges as the master regulator of the entire circuit (Table S6). This is not surprising since previous studies have shown that Oct4/Pou5f1 has the strongest effect on gene expression following its depletion in mESCs, and Oct4/Pou5f1 is the most critical factor for successful iPSC reprogramming [25], [29], [33], [34].

Four new interactions were suggested from the learned model (dark red links in Figure S3). These are the inhibitory interactions from Oct4/Pou5f1 to *Hand1*, *Gata4* and *Ptpn11*, and a negative link from Nr0b1 (Dax1) to *Jarid2*. Hand1 is associated with trophectodermal commitment. The inclusion of an inhibitory link from Oct4/Pou5f1 to *Hand1* is consistent with the trophectodermal phenotype observed after Oct4/Pou5f1 depletion [35]. In addition, it has been reported that Oct4/Pou5f1 binds to *Hand1* and *Gata4* promoters in hESCs [36]. We confirmed the repressive effect of Oct4/Pou5f1 on *Hand1*, *Gata4* and *Ptpn11* expression by depleting Oct4/Pou5f1 using two separate shRNAs followed by RT-PCR (Figure S3). In addition, motif analysis revealed potential Oct4/Pou5f1 binding sites within promoter regions of the three genes (Figure S3). While such data suggest direct regulation by Oct4/Pou5f1 for these genes, it is possible and still consistent with the model, that there are additional unidentified intermediates. Taken together, our results suggest that the learning framework can identify new regulatory relationships that can be experimentally validated. Although the network model topology does not consider protein-protein interactions, the learning process automatically captures functional relationships between multiple pluripotency TFs that are also likely to physically interact. The AND logic operator can indirectly suggest physical binding interactions. Among the 20 known protein-protein interactions that we have collected from prior studies that interconnect the 15 pluripotency regulators, 15 are supported by at least one AND operator (Table S7A). This is a high over-representation compared to random assignments of logic gates (p<0.001, one-tailed Fisher exact test).

Conversely, we rank the protein-protein pairs connected by an AND gate by their co-occurrence frequency in all learned Boolean functions, among the top 10 ranked protein-protein pairs, 6 were previously reported as interaction partners in large-scale interactome studies and 4 were reported in low-throughput studies (Table S7B). Therefore, the optimized network with the learned logic functions is capable of predicting potential protein-protein interactions between TFs. Nevertheless, an AND gate does not necessitate a physical interaction between TFs. Since the top two ranked AND relationships were not supported by direct physical interaction studies, we decided to conduct a co-IP experiment to test one of these, namely the interaction between Nanog and Sox2. Nuclear extract from a mESC line expressing an epitope-tagged Nanog was used to directly test this potential interaction (see Text S1 and Figure S3). The results were negative, suggesting that Nanog and Sox2 may not interact directly. This remains consistent with the model because an AND gate only requires that two or more TFs cooperate to regulate the same target genes by co-binding to promoter or enhancer regions but those factors do not have to physically interact.

### Single and combinatorial in silico perturbations, model dynamics and experimental validations

**Figure 5:**
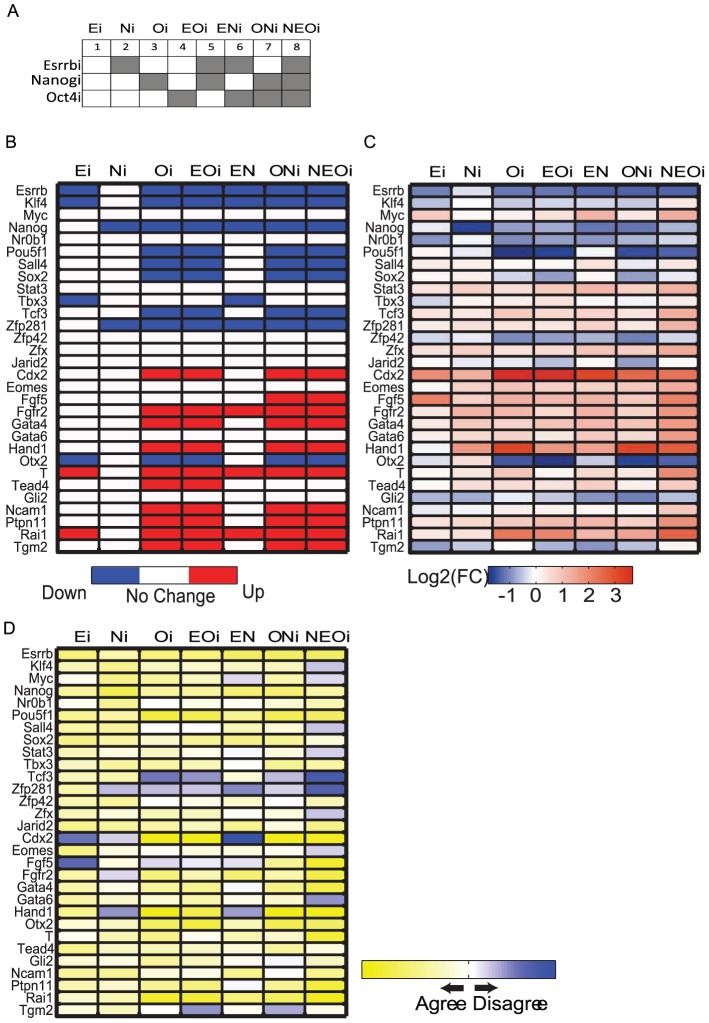
Comparison between computational *in silico* and experimental knockdowns followed by expression measurements. (A) Design of *in silico* and experimental knockdowns. (B) Simulation results of computational knockdowns. Red represents up-regulation, blue represents down-regulation and white represents no change with regards to the unperturbed condition. (C) Results of experimental knockdowns followed by mRNA measurements of all network nodes, where each experiment was repeated twice. Colorbar illustrates log-scaled fold-change. Red represents up-regulation and blue represents down-regulation with regard to the unperturbed condition. (D) Comparison of computational and experimental knockdowns (see Text S1). Colors correspond to discordance score defined in the objective function in the supplementary material. Concordant results are colored in yellow while discordant results are colored in blue with gradients representing the degree of (dis)agreeability.

The refined 30-node network with the learned regulatory logic-gate relationships can be used for dynamical Boolean simulations. However, whether such simulations are predictive requires experimental validation. To this end, we first performed *in silico* and subsequently, experimental knockdowns of Esrrb, Oct4/Pou5f1 and Nanog individually and in all possible double and triple combinations (Figure 5A). The learned ensemble of Boolean dynamical models was used to make predictions about the network response to perturbations. Many Boolean networks consisting of 30 genes/nodes and a set of Boolean functions were sampled randomly from all learned Boolean functions calibrated through the learning workflow. Computational simulations were achieved by forcing a node(s) into a stable OFF state. Starting with 100 random initial condition for each sampled network, step-wise simulations were performed and the resultant stable values for all network nodes (steady-state attractors) were obtained (Figure 5B, Text S1). Technically, simulations were performed using discrete Boolean dynamics with synchronous updating in 30 steps with 10 sampled networks, and 100 random initial conditions. In most cases, steady states were reached within 3 to 9 simulation steps. In the attractor space, on average 83% of the time there was a dominant attractor that is achieved from the 100 random initial conditions. We then performed the same knockdowns in mESCs and measured alkaline phosphatase activities (Figure S4) as well as changes in mRNA and protein levels to verify knockdown efficiencies (Figure S4). Alkaline phosphatase (AP) is a pluripotency stem cell marker, whereas loss of AP activity, as determined by the AP assay, is used to access differentiation of mESCs. We also measured the expression levels of the 30 network genes/nodes using RT-PCR in bulk mESC population, with each experiment performed in duplicate (Figure 5C). To compare the binary vectors from the *in silico* perturbations to continuous experimental Ct-values representing the mRNA levels in the cell population, we calculated post-knockdown gene expression changes relative to the unperturbed condition from both simulations and experiments (Figure 5B–D). A logistic function (see Text S1) was used to compute the consistency between the predicted and measured expression changes, including fold-change magnitudes (Figure 5D, Table S8). Overall, experimental measurements following single or combinatorial knockdowns showed significant agreement with the *in silico* predictions (p-value <10^−15^ compared to a random predictor, see Text S1 for more details) (Figure 5D).

All single and combinatorial *in silico* as well as experimental knockdowns repressed some but not all core pluripotency components and activated selective differentiation markers consistent with published experimental results [25], [37], [38]. In addition, *in silico* simulations showed that knocking down Oct4/Pou5f1 has the most significant effect, in agreement with previous experimental results [25], [29], [33], [34]. However, our dynamical model is unsuccessful in predicting the correct activity values for Cdx2, Fgf5, Tcf3 and Zfp281 for various knockdown conditions. We adopted an alternative strategy to resolve these conflicts by utilizing all data sources to train the model: 1) the initial network topology; 2) the single cell gene expression measurements; and 3) the measurements of the network components after the various knockdowns. The re-learning process resulted in re-wired logic for Cdx2 and Fgf5 that resolved the conflicts for these two nodes (Figure S4G–H). For Tcf3 and Zfp281, the re-learning process did not improve the predictions. It is possible that additional upstream regulators are required to explain these discrepancies. For example, Tcf3 may be differentially regulated by the β-catenin/Wnt signaling pathway in mESCs. Alternatively, Tcf3 and Zfp281 could be differentially regulated in mESCs depleted of Oct4/Nanog/Esrrb. The inconsistent behavior of Tcf3 in the model versus the empirical observations in our experiments is intriguing. The model predicts that knockdowns of Oct4/Nanog/Esrrb would down-regulate *Tcf3*. However, experimental knockdowns resulted in up-regulation of this factor. Increased *Nanog* and *Oct4/Pou5f1* expression after Tcf3 depletion was previously reported [39]. Down-regulation of *Tcf3* following depletion of Oct4/Pou5f1 or Nanog has also been demonstrated [40]. The latter result is consistent with our *in-silico* predictions while the former is not. Training the model with single cell data assumed concordance between mRNA levels and TF activities. For Tcf3 and Zfp281 this assumption may be incorrect. Indeed, in our previous studies after depletion of Nanog, we observed significant discordances between mRNA and encoded protein levels [41]. In addition to the Nanog, Oct4/Pou5f1 and Esrrb single and combinatorial knockdowns we also depleted Jarid2 and observed significant agreement between predicted and experimental results (Figure S4).

Next, we asked if prediction accuracy is mostly the result of calibrating the initial network topology with the single cell data, or if it is already largely embedded in the topology extracted from the ESCAPE database, with the single cell data contributing only minor tuning. To address this question, we randomly and sequentially flipped binary single cell gene expression values or, similarly reassigned network links from ESCAPE (see Text S1). We demonstrate this methodology using a toy network with simulated data (Figure S7). Given any directed network and single cell gene expression data (Figure S7), the regulatory logic can be learned. Then, when *in-silico* knockdowns are performed, computational knockdowns can be compared with experimental knockdowns. Each entry in the comparison heatmap was calculated as 1 – discordance-score. Note that the score didn't reach 100 despite the ideal example, due to the logistic function used. Both shuffling single cell values, or links from the original topology of the network, reduced the predictive power of the model to approximately equal extents, demonstrating that both sources of data contribute to prediction accuracy (Figure S4).

**Figure 6:**
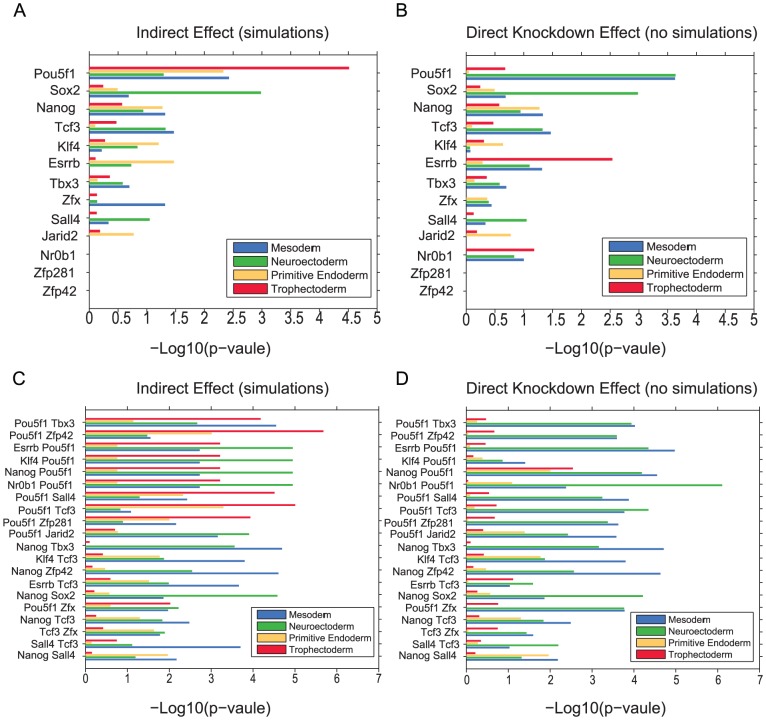
Computational *in silico* knockdowns of all possible single and double perturbations linked to predicted lineage differentiation outcomes. (A, C) Predicted lineage specification based on dynamical model simulations. (B, D) Lineage predictions based on direct effects of knockdowns. Predictions are based on differentially expressed genes from LOF studies and promoter binding of transcription factors to differentially expressed genes based on mESC ChIP-chip/seq studies from the ESCAPE database.

Because the predictions obtained from the dynamical model are generally reliable, we simulated all possible single or combinatorial knockdowns to predict mESC lineage commitment outcomes (Figure 6, Text S1). The 15 lineage-specific markers in the network may be limited and biased because lineage specification involves more genes. Therefore, we linked the simulated knockdowns to a larger set of 40 lineage marker genes (Text S1). We compared predictions that are based on the resultant state of the network core components after simulated knockdowns, with predictions that are based solely on the additive effect of the direct targets of individual and combinations of TFs without simulations. Interestingly, predictions made through the Boolean modeling approach were more consistent with lineage commitment knowledge compared with the direct TF target-based predictions; for example, forcing Oct4/Pou5f1 into a stable OFF state in our dynamical model results in more pronounced predicted trophectodermal differentiation when compared with predictions that are based solely on Oct4/Pou5f1 target genes. Trophectoderm induction after Oct4/Pou5f1 depletion is well-established [42]. We also examined lineage marker gene levels after depleting Esrrb or Jarid2. For Esrrb, simulation-based predictions were more consistent with experimental data showing greater effects on neuroectodermal than trophectodermal or mesodermal differentiation. In the case of Jarid2, both simulation-based and direct targets lineage predictions were consistent with our experimental observations which point to primitive endoderm differentiation (Figures 6 and S5). Overall, the Boolean model appears to resolve indirect effects of TFs on lineage commitment. The model may be most useful for prioritizing combinations of knockdowns not easily tested in high-throughput.

### Consensus and unique interactions across four learned models and two culture conditions

Finally, we constructed two additional dynamical models learned from two recently published single cell gene expression datasets collected from CCE and Nanog removed (Nanog_R) mESCs using the same Fluidigm platform in the same laboratory [43]. To compare the four dynamical models constructed from the various types of mESCs we counted unique and shared regulatory interactions (Figure S6). Interestingly, we identified 41 unique interactions present only in one model under one condition, and 13 consensus interactions shared by all four models and conditions. In addition, among all the learned interactions from the four models, there are 42 interactions that appear in 3 out of 4 models, and 62 interactions that are found in 2 out of 4 models. Hence, on average each model contains ∼10 unique interactions and ∼40 interactions shared by at least one another model. One of the consensus interactions is Nanog regulation of *Zfp281*, reinforcing the importance of this interaction. Likewise, Oct4/Pou5f1 consistently positively regulates *Zfp42/Rex1* and *Sall4*. Intriguingly, the positive feedback loop between Oct4/Pou5f1 and Sox2 exists in all models in serum/LIF but not in 2i/LIF, suggestive of culture-specific regulatory interactions. Other differences can stem from cell-line differences, differences in the times the experiments were conducted, as well as instrument noise. Overall we consider the agreement among all 4 models to be relatively robust.

### Conclusions and summary

For this study we first developed a directed and signed network consisting of 15 pluripotency and self-renewal regulators connected to 15 lineage marker genes. We then learned the underlying regulatory logic among network components utilizing single cell gene expression data. Gene expression in single cells uncovered some differences in mESCs cultured under serum/LIF versus 2i/LIF conditions. Characterizing such culture-dependent differences is important for understanding the enhanced iPSC reprogramming efficiency in the 2i/LIF condition [44]. Expression in single cells was found to be mostly bimodal and this fits well with a Boolean modeling framework. The utility of such Boolean modeling approach is the ability to perform predictions of network behavior after *in silico* perturbations, and the ability to control and constrain the free parameter space. The consistency such *in silico* perturbations were compared to experimental combinatorial shRNA perturbations. The good agreement between the model and the experimental validation suggest that the model does capture some of the real dynamics of the pluripotency and self-renewal circuit. Nevertheless, many challenges remain. Our model is binary, with genes and their products merged into single nodes assuming a direct correlation between TF activity and mRNA expression. We observed such correlations for Nanog, Oct4/Pou5f1 and Esrrb (Figure S4), but for other TFs this may not be the case. Furthermore, while there is ample evidence describing how pluripotency TFs regulate lineage-specific genes, little is known about lineage regulator-mediated suppression of the pluripotency circuit. In addition, gene expression is controlled by protein complexes and epigenetic modifications not explicitly incorporated into the Boolean model. Integration of TFs, histone modifications and DNA methylation may result in a more complex model but also such model will be more accurate and revealing [45]. Nevertheless, rapid progress in the field indicates that we will gradually be able to obtain more refined and dynamic view of pluripotency, self-renewal, lineage-specific commitment and differentiation, as well as better understand the process of iPSC reprogramming. Such views will enable the realization of pluripotent cell-based applications in regenerative medicine.

## Methods

### Cell culture and single cell RT-PCR analysis

The Esrrb_R rescue cell line was derived from AINV-15 ESCs and cultured as previously described [25] in ESC media containing doxycycline (1 µg ml-1 Sigma). CCE mESCs are one of the first established stem cell lines [46]. They are derived from the 129/Sv mouse strain. All cells used in these experiments were under passage 80. Serum-free ESC cultures were performed as previously described [47], [48]. Briefly, cells were maintained without feeders in serum-free N2B27 media prepared as described [47] and supplemented with LIF (Chemicon, Millipore) and 2i inhibitors [18]. The two inhibitors (Stemgent) block GSK3β, (CHIR99021; 3 µM) and MEK1/2 (PD0325901; 1 µM). All cultures were maintained at 37°C with 5% CO_2_. Inventoried TaqMan assays (20×, Applied Biosystems) were pooled to a final concentration of 0.2× for each of the 96 assays. Single Esrrb_R cells expressing both GFP and SSEA-1 were FACS-sorted directly into 10 µL RT-PreAmp Master Mix (5.0 µL CellsDirect 2× Reaction Mix, 2.5 µL 0.2× assay pool, 0.2 µL SuperScript III RT/Platinum Taq Mix from the (CellsDirect One-Step qRT PCR Kits, Invitrogen) and 1.3 µL TE buffer. Cell lysis and gene-specific reverse transcription were performed at 50°C for 20 min. Reverse transcriptase was heat-inactivated for 2 min at 95°C. Subsequently, single cell cDNA was pre-amplified using a multiplexed, target-specific amplification protocol (denaturation at 95°C for 15 sec, and annealing and amplification at 60°C for 4 min for a total of 18 cycles). Pre-amplified products were diluted 5-fold prior to amplification using a Universal PCR Master Mix and inventoried TaqMan gene expression assays (Applied Biosystems) in 96×96 Dynamic Arrays on a BioMark System (Fluidigm). Amplification included a 10 min, 95°C hot-start followed by 40 cycles of a two-step program consisting of 15 sec at 95°C and 60 sec at 60°C. Ct-values were calculated using BioMark Real-Time PCR Analysis Software v2.0 (Fluidigm). Values greater than 35 were considered non-detectable and recorded as 35.

### Short hairpin (sh)RNA design and plasmid construction

Gene-specific 19nt shRNAs were designed based on a previously described algorithm using an in-house Perl script [49]. All shRNA sequences were BLASTed to ensure specificity. Synthesized oligomers were annealed and ligated into the pSuper.puro vector (Oligoengine). To make the Oct4/Nanog/Esrrb combinatorial shRNA constructs, ClaI-XhoI sites were used to insert H1-shRNA cassettes digested with BstBI-XhoI. The shRNA encoding sequences are: Oct4– GAAGGATGTGGTTCGAGTA (shRNA_#1) and GCGAACTAGCATTGAGAAC (shRNA_#2), Nanog– GAACTATTCTTGCTTACAA, Esrrb– GATTCGATGTACATTGAGA and Jarid2 – TCACTGTCCTCCCAAATAA.

### Cell culture and transfection

Mouse CCE ESCs were cultured feeder-free on 0.1% gelatin-coated plates in ESC media (Dulbecco's modified Eagle's medium (DMEM; Hi-Glucose), 15% fetal bovine serum, non-essential amino acids, L-glutamine, β-mercaptoethanol, penicillin/streptomycin, sodium pyruvate and LIF (Millipore). Serum was purchased from HyClone. This serum is embryonic stem cell qualified and therefore does not require heat inactivation. The specific lot of serum was rigorously tested to ensure robust self-renewal with little spontaneous differentiation as assessed by mESC morphologies and alkaline phosphatase staining. All cell cultures were maintained at 37°C with 5% CO_2_. Gene-specific or scrambled shRNA constructs, the GFP-shRNA construct and empty vector (all 3 ug) were transfected using Lipofectamine 2000 (Invitrogen) according to the manufacturer's instructions. Transfected cells were selected for 48 hrs. in puromycin (1.5 ug/ml). Mock transfections resulted in no surviving cells after selection.

### Real-time quantitative PCR

Total RNA was Trizol-extracted (Invitrogen), column-purified with RNeasy kits (Qiagen), and reverse transcribed using the High Capacity reverse transcription kit (Applied Biosystems). All quantitative PCR analyses were performed using the Fast SYBR Green Master Mix (Applied Biosystems) following the manufacturer's protocol on the LightCycler480 Real-Time PCR System (Roche). Each PCR reaction generated a specific amplicon, as demonstrated by melting-temperature profiles (Dissociation Curve analysis). No PCR products were observed in the absence of template. Data were normalized to *Gapdh* and represented relative to empty-vector transfected controls. Primer sequences are available in Table S9.

### Western blot analysis

Cells were scraped/trypsinized, washed in PBS and incubated for 20 min in cold RIPA buffer without SDS. Protein concentrations were determined using Bradford Dye (Bio-Rad). Total proteins (∼10 ug) were separated on SDS–PAGE gels and transferred to PVDF membranes (Millipore). Membranes were probed with specific primary antibodies followed by HRP-conjugated secondary antibodies and developed with ECL (Amersham). Primary antibodies were: Oct4 (sc9081, Santa Cruz), Nanog (A300-398A, Bethyl), Actin (sc1615, Santa Cruz), Sox2 (sc17320, Santa Cruz) and Esrrb (300–748, Novus Biologicals). Amido-black was used to detect core histones. Quantification of protein bands was performed using Adobe Photoshop. Relative protein level differences were calculated by normalization to actin levels and shown relative to empty-vector transfected sample.

### Alkaline phosphatase staining assay

An alkaline phosphatase detection kit (Stemgent) was used to measure activity according to the manufacturer's instructions.

### Signed directed network assembly

From the ESCAPE database, a network containing 15 core pluripotency and 15 lineage-specific components was extracted. Arrows were established if there was evidence for binding of a specific TF to a target gene from mESC ChIP-chip/seq studies as well as a change in target gene expression level after loss-of-function (LOF) or gain-of-function (GOF) of the same TF in mESCs. We applied a majority-voting function giving more weight to LOF than to GOF evidence and binarized the output as either activation (+1) or inhibition (−1) using the sign function:
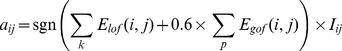
(1)


Where 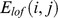 is 1 for activation or −1 for inhibition according to the 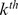 LOF study where gene 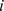 is depleted by RNAi or deleted by homologous recombination. 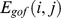 is 1 for activation or otherwise −1 for inhibition according to the 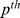 GOF study where gene 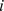 is over-expressed. 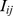 C with value 1 if there exists at least one protein-target gene promoter binding interaction connecting transcription factor 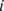 to target gene 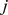 from ChIP-chip/seq studies and 0 otherwise. A few links (8 out of 450 potential interactions) were manually refined in cases with contradictory evidence using information from small-scale functional studies (Table S2).

### K-means clustering

Experimental data from the Fluidigm platform measure transcript abundance of up to 96 genes analyzed in 96 single cells. Ct-values were normalized to the housekeeping gene *Gapdh* levels in each cell and converted to binary values (1 for high expression and 0 for low expression) using K-means clustering with K = 2 (MATLAB, Bioinformatics Toolbox). Histogram curves for normalized Ct-values were smoothened using the Kernel smoothing algorithm (MATLAB, Statistics Toolbox). Hierarchical clustering for binarized and continuous expression levels of the 30 network genes in 96 individual cells were hierarchically clustered using the average-linkage clustering algorithm (MATLAB, Bioinformatics Toolbox).

### Dynamical simulations and comparison between *in silico* and experimental knockdowns

The calibrated network inferred from the Boolean-function-learning-process is simulated using discrete Boolean dynamics with synchronous updating and the learned Boolean functions. An expression pattern 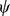 is defined as a state vector 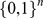 where 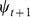 is determined by 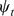 with the underlying Boolean functions as follows: 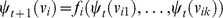. One Boolean network consisting of 30 genes and a set of Boolean functions is sampled randomly from all learned Boolean functions calibrated through the learning workflow. For each *in silico* simulation setting, 10 networks were sampled and the gene status (0 or 1) is recorded in 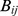 for each gene 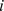 in condition 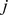 as follows: for each sampled network 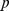, 100 random initial conditions are evolved for 30 steps in synchronous mode. We set the step of 30 since all networks can reach steady states within a step of 30 in our case. *In silico* knockdowns are achieved by forcing gene(s) to the ‘OFF’ state all the time while recording the final state of the Boolean network. Each final state, as denoted by an attractor 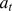 is recorded with weight 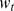 based on the size of its basin (defined as the set of initial states that lead to an attractor). Since a network can reach multiple steady states with certain probabilities, we weighted each stable state 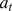 by the probability of being in that particular state 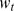. Thus the final binary state of each gene 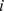 in each condition 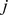 for a sampled network 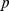 is determined by 
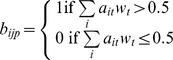
, where 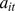 denotes the state of gene 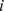 in attractor 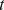 and 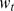 is the weight of attractor 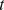. Then 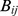 for 10 sampled networks is calculated as follows:
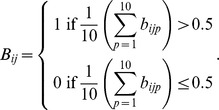



The formula for calculating the discordance score 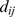 for gene 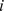 in condition 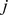 between simulation and experiment is as follows: 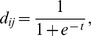


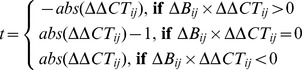



Where 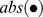
is absolute value function; 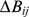 reflects the expression change for gene 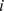 in condition 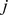 relative to unperturbed condition under *in silico* simulations, with values from 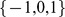 representing ‘down-regulation’, ’no-change’ and ‘up-regulation’, respectively; 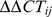 reflects 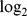 fold-change of gene expression in knockdown mESCs relative to empty-vector transfected controls measured by RT-PCR, with negative values representing ‘up-regulation’ and positive values representing ‘down-regulation’. As the logistic function is monotonically increasing, larger value of 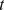 would result in larger discordance score.

### Quantifying agreement between experimental results and simulations results of networks learned from randomized single cell data/network topology

Single cell expression data are gradually randomized by flipping data points with a certain percentage 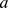. The topology of the network is randomized by rewiring 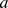 percent links represented as entries in the adjacency matrix underlying the 30 node network extracted from the ESCAPE database. An objective function 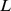 is defined to quantify the error between *in silico* knockdown simulations and the experimental results. 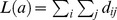, where 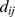 is the same discordance score defined above. Relative accuracy is defined as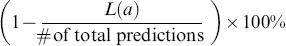. For each 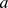, permutation level and learning process is repeated 10 times to obtain means and standard deviations.

### Statistical significance of model prediction accuracy

Mean discordance scores across all genes and perturbation conditions (30×7 = 210) from the model were compared to prediction results with a random predictor. This is a simple predictor with outputs randomly chosen from −1, 0 or +1 representing ‘down-regulation’, ‘no change’ or ‘up-regulation’, respectively. A total of 500 random M_30×7_ matrices were generated using the random predictor. Rows represent genes and columns represent perturbation conditions. Individual mean discordance scores were calculated for each of the 500 random matrices. A one-sample t-test was performed to test the null hypothesis that the random sample mean is equal to mean discordance scores from the model.

## Supporting Information

Figure S1 Summary of feedback loops.
(PDF)

Figure S2 Characterization of gene expression of mESCs in serum/LIF and 2i/LIF at the single cell resolution.
(PDF)

Figure S3 Details of ensemble of learned networks and validation of novel interactions identified through the logic learning process.
(PDF)

Figure S4 Validation of knockdowns.
(PDF)

Figure S5 Expression measurements of lineage specification markers after knockdowns in mESCs.
(PDF)

Figure S6 Ensemble and comparison of learned networks from single cell datasets.
(PDF)

Figure S7 Illustration of quantifying agreement between simulation and experiment results.
(PDF)

Table S1 Literature evidence and processing methods for extraction of interactions.
(PDF)

Table S2 References for modifications of interactions from low-throughput studies.
(PDF)

Table S3 Information about probes/genes used in the single cell Fluidigm experiments.
(PDF)

Table S4 Boolean function distribution and degeneracy counts.
(PDF)

Table S5 Comparing expression levels for genes in serum/LIF vs 2i/LIF.
(PDF)

Table S6 Out-degree centrality measures in terms of critical links of nodes in the ensemble, representative networks of mESCs in serum/LIF and 2i/LIF.
(PDF)

Table S7 Relationship between protein pairs connected by ‘AND’ gate and literature evidence.
(PDF)

Table S8 Comparison values of computational and experimental knockdowns used in Figure 5D.
(PDF)

Table S9 Primers used for RT-PCR analysis in mESCs.
(PDF)

Text S1 Supporting information text including details about learning and optimization of Boolean transition functions, analysis of Oct4/Pou5f1 binding sites within gene promoter regions, comparison of distribution of Esrrb expression in Esrrb-rescue mESCs and other single mESCs, dynamical simulations and comparison between *in silico* and experimental knockdowns, quantifying agreement between experimental results and simulations results of networks learned from randomized single cell data/network topology, defining large sets of lineage-specific signature genes, lineage commitment predictions, and co-immuno-precipitation validation of Nanog-Sox2 interaction in ESCs.
(PDF)
