# Supplementary material for: Construction and Validation of a Regulatory Network for Pluripotency and Self-Renewal of Mouse Embryonic Stem Cells
Source: PLoS Comput Biol. 2014 Aug 14;10(8):e1003777. doi: 10.1371/journal.pcbi.1003777 (PMC4133156; doi:10.1371/journal.pcbi.1003777)
Supplement: Figure S1 — Summary of feedback loops. (PDF) [file pcbi.1003777.s001.pdf]

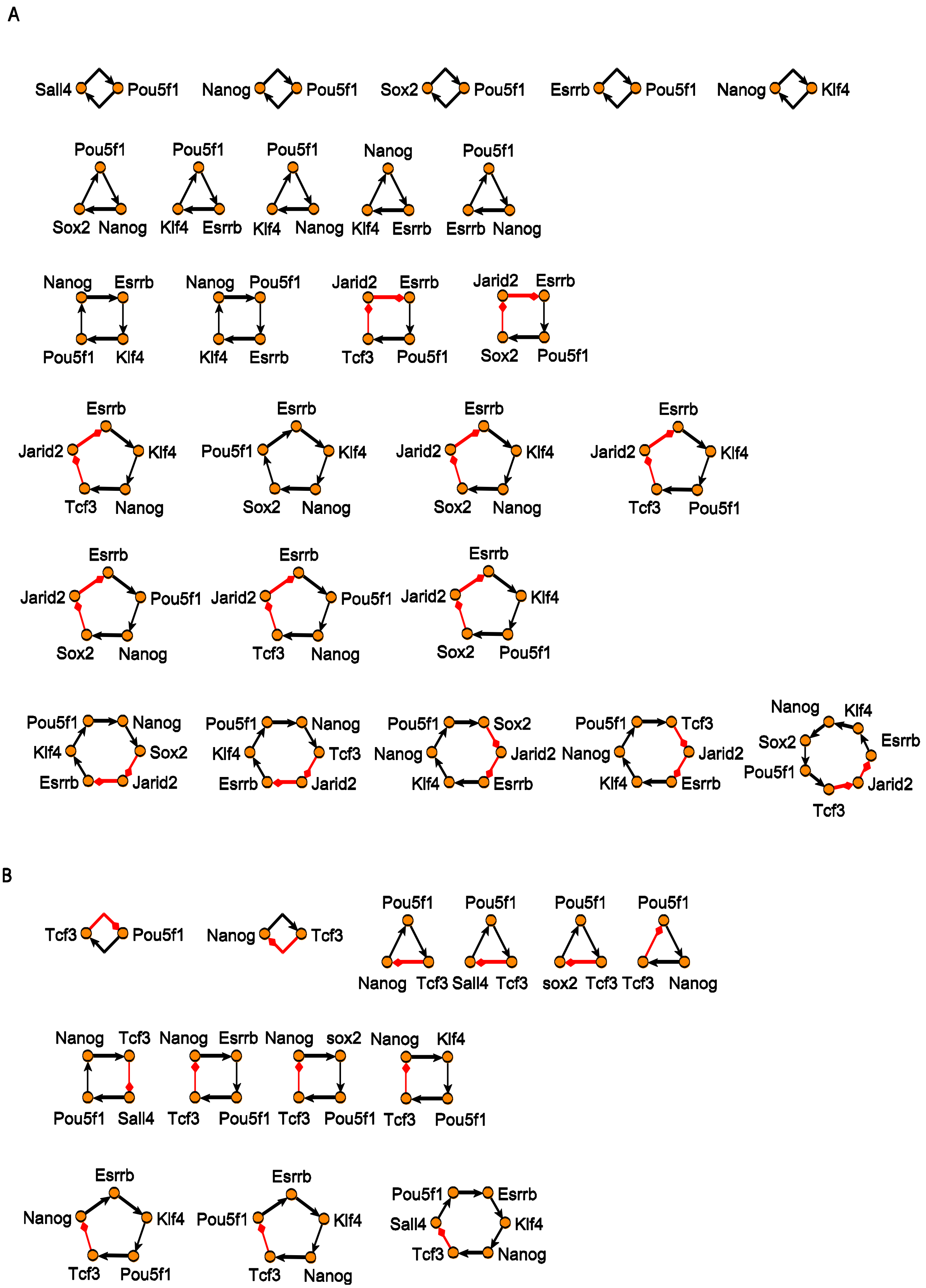

Figure S1. Summary of feedback loops

Enumeration of (A) positive and (B) negative feedback loops in the pre-learning network. Black edges with standard arrowhead indicate activation; red edges with square arrowhead indicate repression.
