# Supplementary material for: Construction and Validation of a Regulatory Network for Pluripotency and Self-Renewal of Mouse Embryonic Stem Cells
Source: PLoS Comput Biol. 2014 Aug 14;10(8):e1003777. doi: 10.1371/journal.pcbi.1003777 (PMC4133156; doi:10.1371/journal.pcbi.1003777)
Supplement: Figure S3 — Details of ensemble of learned networks and validation of novel interactions identified through the logic learning process. (PDF) [file pcbi.1003777.s003.pdf]

A

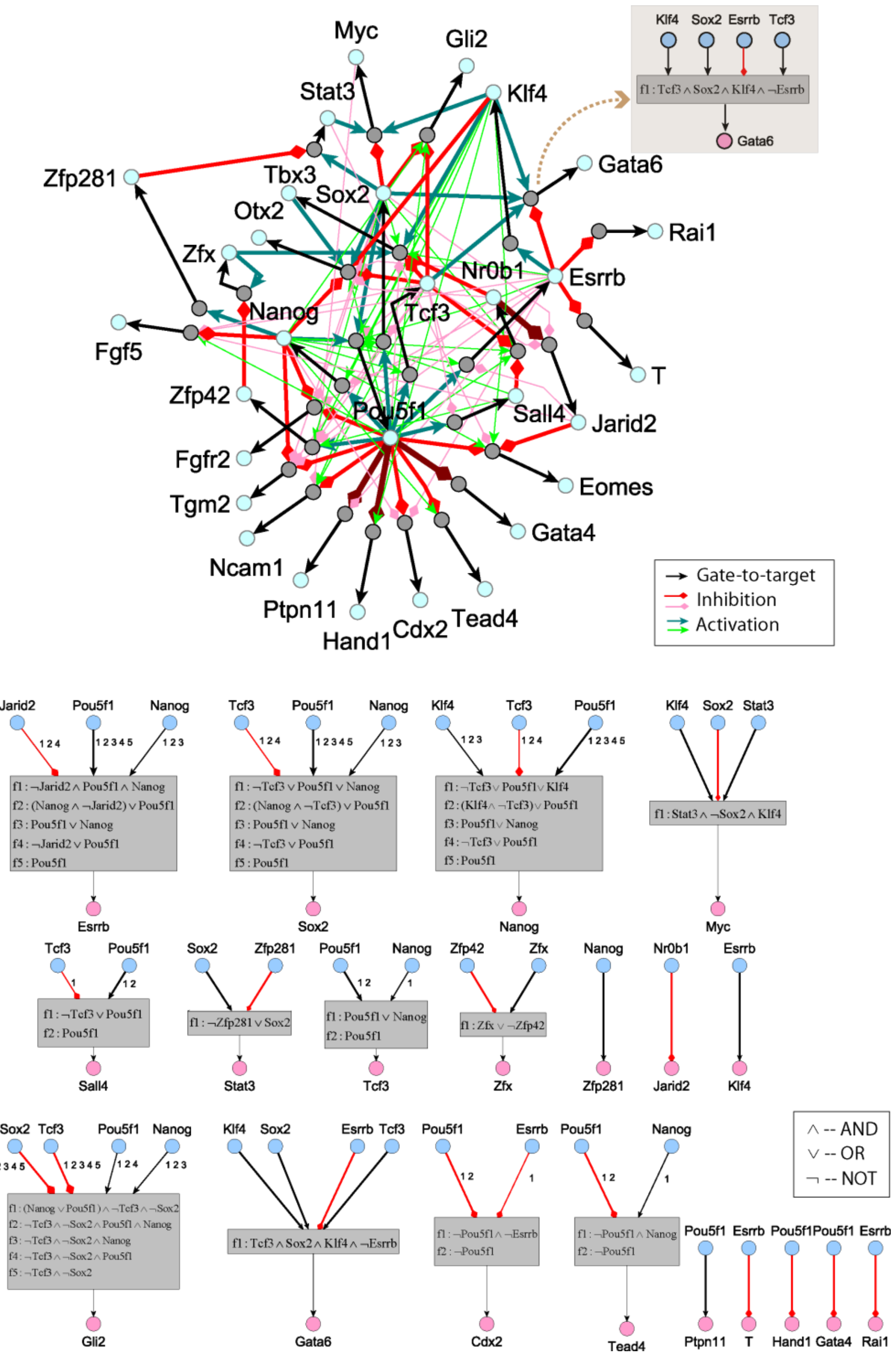

Myc
Gli2
Stat3
Klf4
Zfp281
Tbx3
Sox2
Otx2
Gata6
Rai1
Zfx
Nr0b1
Esrrb
Nanog
Tcf3
Fgf5
T
Zfp42
Pou5f1
Sall4
Jarid2
Fgfr2
Eomes
Tgm2
Gata4
Ncam1
Ptpn11
Hand1
Cdx2
Tead4
Klf4 Sox2 Esrrb Tcf3
f1 : Tcf3 ∧ Sox2 ∧ Klf4 ∧ ¬Esrrb
Gata6
Gate-to-target
Inhibition
Activation
B
Jarid2 Pou5f1 Nanog
f1 : ¬Jarid2 ∧ Pou5f1 ∧ Nanog
f2 : (Nanog ∧ ¬Jarid2) ∨ Pou5f1
f3 : Pou5f1 ∨ Nanog
f4 : ¬Jarid2 ∨ Pou5f1
f5 : Pou5f1
Esrrb
Tcf3 Pou5f1 Nanog
f1 : ¬Tcf3 ∨ Pou5f1 ∨ Nanog
f2 : (Nanog ∧ ¬Tcf3) ∨ Pou5f1
f3 : Pou5f1 ∨ Nanog
f4 : ¬Tcf3 ∨ Pou5f1
f5 : Pou5f1
Sox2
Klf4 Tcf3 Pou5f1
f1 : ¬Tcf3 ∨ Pou5f1 ∨ Klf4
f2 : (Klf4 ∧ ¬Tcf3) ∨ Pou5f1
f3 : Pou5f1 ∨ Nanog
f4 : ¬Tcf3 ∨ Pou5f1
f5 : Pou5f1
Nanog
Klf4 Sox2 Stat3
f1 : Stat3 ∧ ¬Sox2 ∧ Klf4
Myc
Tcf3 Pou5f1
f1 : ¬Tcf3 ∨ Pou5f1
f2 : Pou5f1
Sall4
Sox2 Zfp281
f1 : ¬Zfp281 ∨ Sox2
Stat3
Pou5f1 Nanog
f1 : Pou5f1 ∨ Nanog
f2 : Pou5f1
Tcf3
Zfp42 Zfx
f1 : Zfx ∨ ¬Zfp42
Zfx
Nanog
Zfp281
Nr0b1
Jarid2
Esrrb
Klf4
Sox2 Tcf3 Pou5f1 Nanog
f1 : (Nanog ∨ Pou5f1) ∧ ¬Tcf3 ∧ ¬Sox2
f2 : ¬Tcf3 ∧ ¬Sox2 ∧ Pou5f1 ∧ Nanog
f3 : ¬Tcf3 ∧ ¬Sox2 ∧ Nanog
f4 : ¬Tcf3 ∧ ¬Sox2 ∧ Pou5f1
f5 : ¬Tcf3 ∧ ¬Sox2
Gli2
Klf4 Sox2 Esrrb Tcf3
f1 : Tcf3 ∧ Sox2 ∧ Klf4 ∧ ¬Esrrb
Gata6
Pou5f1 Esrrb
f1 : ¬Pou5f1 ∧ ¬Esrrb
f2 : ¬Pou5f1
Cdx2
Pou5f1 Nanog
f1 : ¬Pou5f1 ∧ Nanog
f2 : ¬Pou5f1
Tead4
Pou5f1 Esrrb Pou5f1 Pou5f1 Esrrb
Ptpn11 T Hand1 Gata4 Rai1
∧ -- AND
∨ -- OR
¬ -- NOT

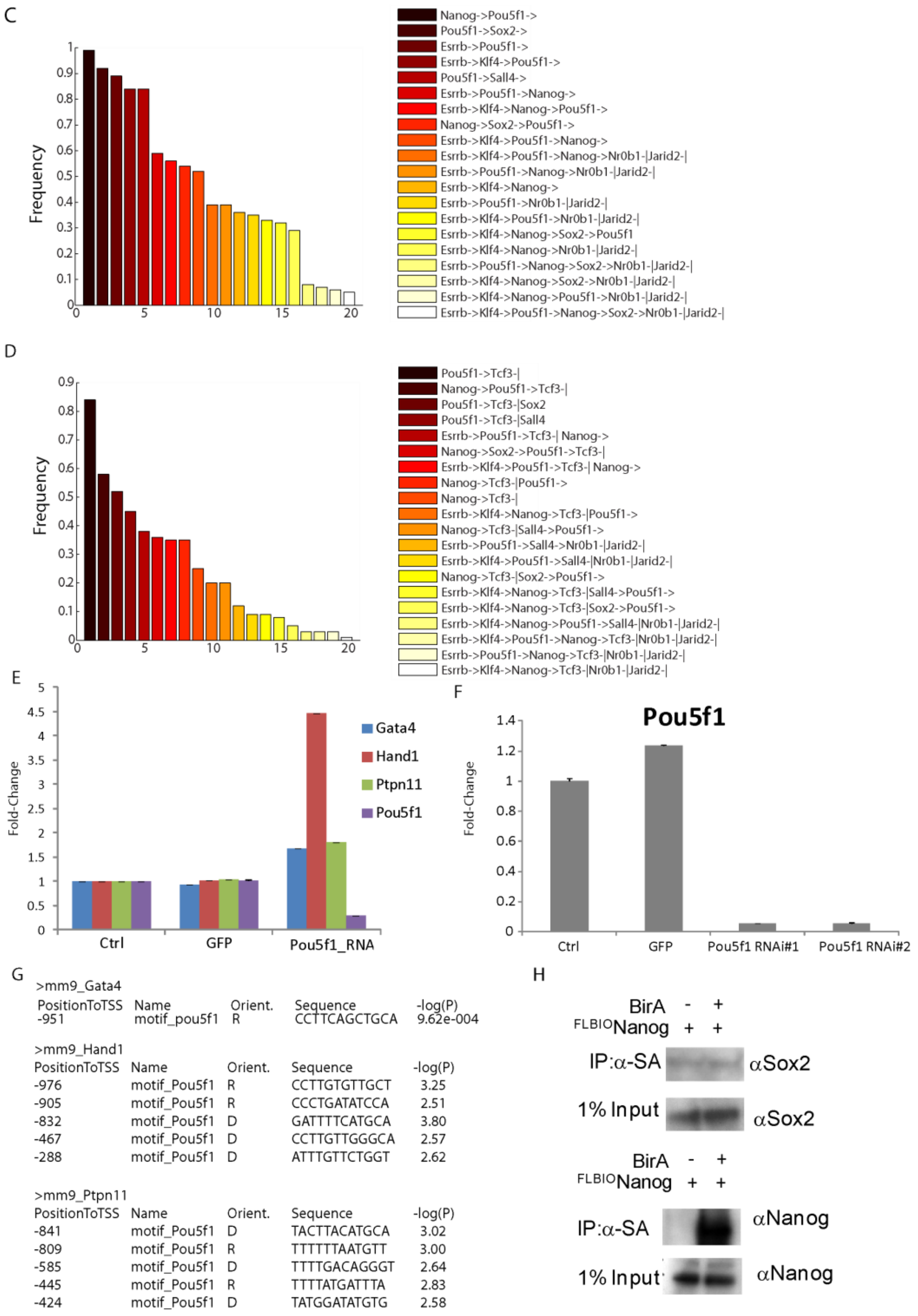

Figure S3. Details of ensemble of learned networks and validation of novel interactions identified through the logic learning process

(A) The network with learned logic consisting of 30 genes/proteins and inferred Boolean functions learned from the serum/LIF single cell data. Light cyan nodes represent genes; gray squares represent learned regulatory logic (as gates) with the inset shadowed box exemplifies one such learned transition function set upstream of *Gata6*. Links from upstream parent nodes appearing in more than 90% of equally well fitted Boolean functions are defined as 'master links' and highlighted in dark green (activation) and red (repression). Other links are colored in light green (activation) and pink (inhibition). The four novel links are highlighted in dark red. (B) Selected examples of transition Boolean functions for 20 genes. Diamond heads are links from repressors whereas arrowheads are links from activators. Arrow thickness is proportional to the probability of upstream regulators to be involved in the best fitted Boolean functions. Arrow labels index the Boolean function. The sign $\neg$represents the logic operator 'NOT', $\wedge$ represents 'AND' and $\vee$ represents 'OR'. Boolean functions for all genes are available in Supplementary Files 5-7. (C-D) Occurrence of individual positive (C) and negative (D) feedback loops in 100 sampled post-learning Boolean networks. -> indicates activation and -| indicates repression. The sign of the link from the end node to start node is indicated by the symbol in the end of each legend. (E) Fold-changes

of gene expression of control and Oct4/Pou5f1-knockdown mESCs. ‘Ctrl’ represents empty-vector injected mESCs, ‘GFP’ represents GFP-vector injected mESCs and ‘Pou5f1_RNAi’ represents Oct4/Pou5f1-shRNA injected mESCs. (F) Fold-changes of *Oct4/Pou5f1* expression of control and different shRNA knockdown mESCs. ‘Ctrl’ represents empty-vector injected mESCs, ‘GFP’ represents GFP-vector injected mESCs, “Pou5f1_RNAi#1” represents shRNA#1 injected mESCs and “Pou5f1_RNAi#2” represents shRNA#2 injected mESCs. (G) Potential binding sites for Oct4/Pou5f1 within the promoter region (-1kb from TSS) of *Gata4*, *Hand1*, *Ptpn11*. (H) Co-IP experiment testing Nanog-Sox2 interaction in mESCs nuclear extract from NGA2 (left lane) and NGB19 ESCs (right lane) were used for SA capture followed by western blotting with anti-Sox2 (upper) and Nanog (lower). NGA2 ESCs express Nanog proteins with Flag and biotin dual tag in Nanog null ESCs. The BirA transgene expressing biotin ligase was engineered into NGA2 ESCs to obtain NGB19 ESCs. The result from this experiment shows that we could not detect a physical interaction between these factors.
