# Supplementary material for: Construction and Validation of a Regulatory Network for Pluripotency and Self-Renewal of Mouse Embryonic Stem Cells
Source: PLoS Comput Biol. 2014 Aug 14;10(8):e1003777. doi: 10.1371/journal.pcbi.1003777 (PMC4133156; doi:10.1371/journal.pcbi.1003777)
Supplement: Figure S4 — Validation of knockdowns. (PDF) [file pcbi.1003777.s004.pdf]

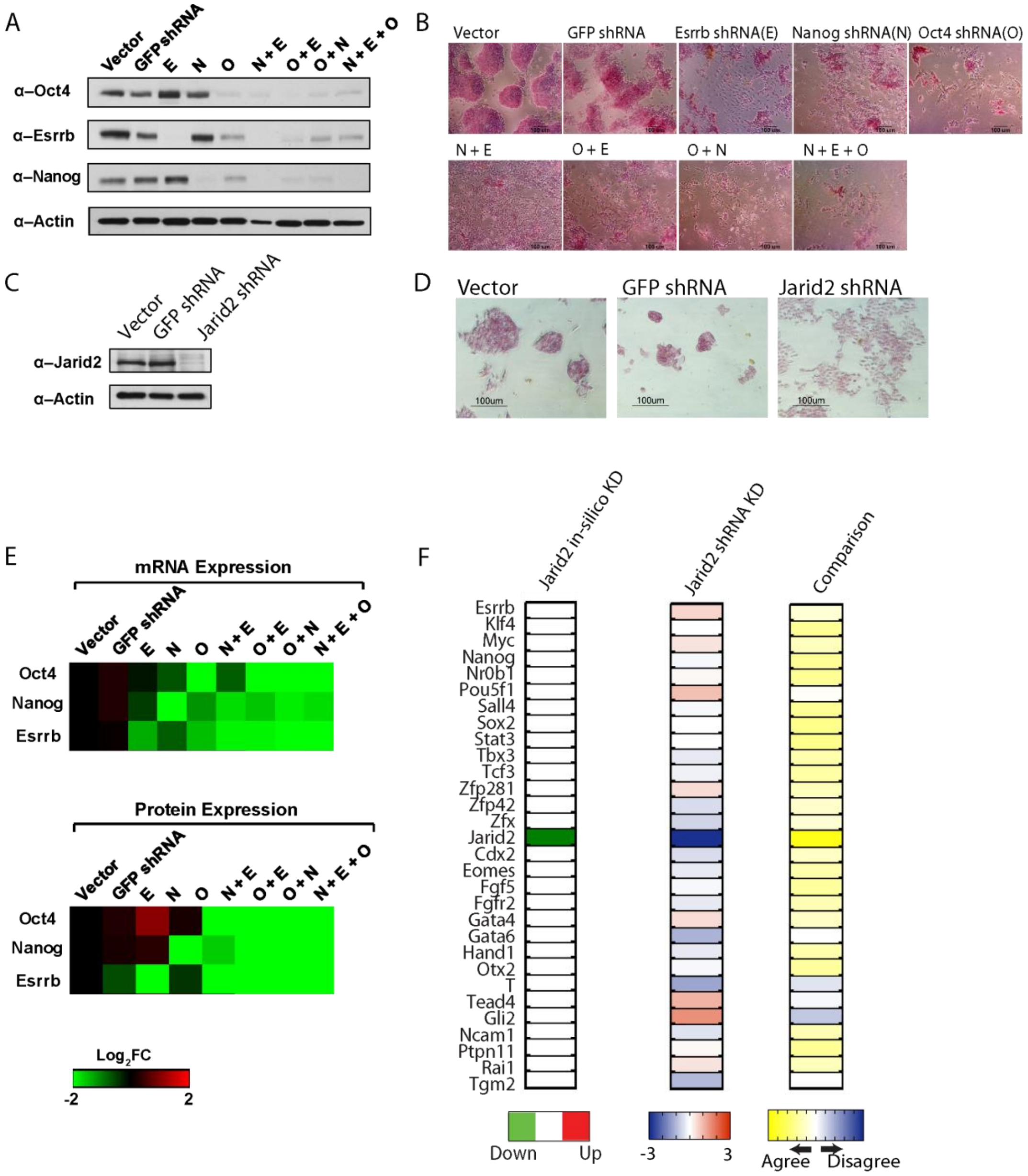

A
Vector
GFP shRNA
E
N
O
N+E
O+E
O+N
N+E+O
α–Oct4
α–Esrrb
α–Nanog
α–Actin
B
Vector
GFP shRNA
Esrrb shRNA(E)
Nanog shRNA(N)
Oct4 shRNA(O)
N + E
O + E
O + N
N + E + O
C
Vector
GFP shRNA
Jarid2 shRNA
α–Jarid2
α–Actin
D
Vector
GFP shRNA
Jarid2 shRNA
100um
E
mRNA Expression
Oct4
Nanog
Esrrb
Protein Expression
Log2FC
-2
2
F
Jarid2 in-silico KD
Jarid2 shRNA KD
Comparison
Esrrb
Klf4
Myc
Nanog
Nr0b1
Pou5f1
Sall4
Sox2
Stat3
Tbx3
Tcf3
Zfp281
Zfp42
Zfx
Jarid2
Cdx2
Eomes
Fgf5
Fgfr2
Gata4
Gata6
Hand1
Otx2
T
Tead4
Gli2
Ncam1
Ptpn11
Rai1
Tgm2
Down
Up
-3
3
Agree
Disagree

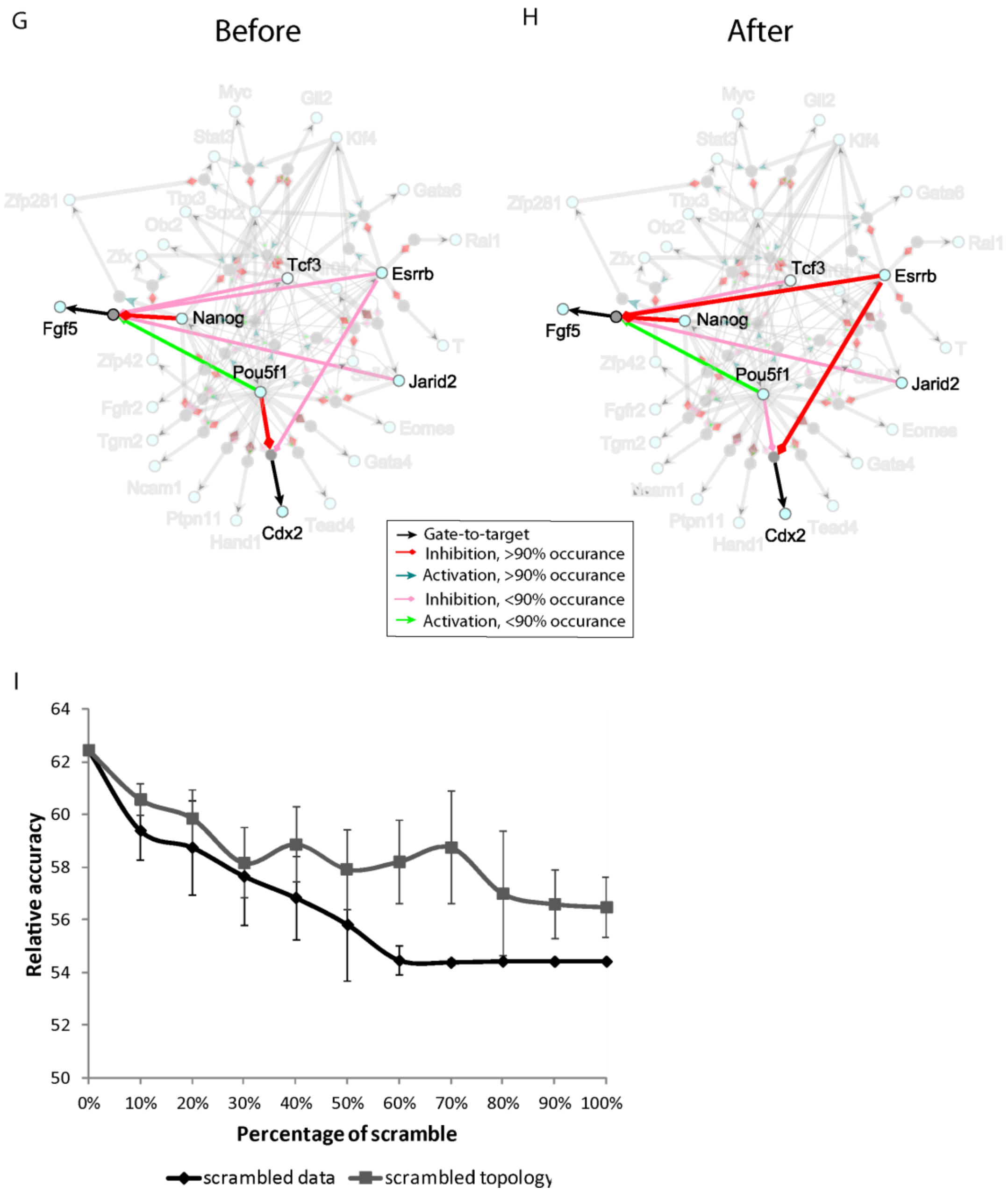

Figure S4. Validation of knockdowns

(A,C) Western blot of (A) Oct4/Pou5f1, Esrrb, Nanog and (C) Jarid2 with Actin as control. (B,D) Alkaline phosphatase staining after two days knockdowns with shRNAs. (E) Comparison between mRNA and protein levels of Oct4/Pou5f1, Nanog and Esrrb in regular and knockdown mESCs. (F) Comparison of *in silico* and experimental knockdown of Jarid2. Simulation (left), experimental (middle) and comparison (right) results of Jarid2 knockdowns. (G-H) Differential links regulating *Fgf5* and *Cdx2* before (G) and after (H) relearning using the RT-PCR knockdown data. (I) Percentage of input data points flipped, or entries in the adjacency matrix of the network scrambled, plotted against relative accuracy of comparing simulations from learned Boolean models calibrated by the single cell data and the initial topology, with experimental knockdown results. Black line represents results of flipped expression data points from the binarized single cell data with intact network topology, grey line represents results of scrambled network wiring with intact single cell data.
