# Supplementary material for: Construction and Validation of a Regulatory Network for Pluripotency and Self-Renewal of Mouse Embryonic Stem Cells
Source: PLoS Comput Biol. 2014 Aug 14;10(8):e1003777. doi: 10.1371/journal.pcbi.1003777 (PMC4133156; doi:10.1371/journal.pcbi.1003777)
Supplement: Figure S5 — Expression measurements of lineage specification markers after knockdowns in mESCs. (PDF) [file pcbi.1003777.s005.pdf]

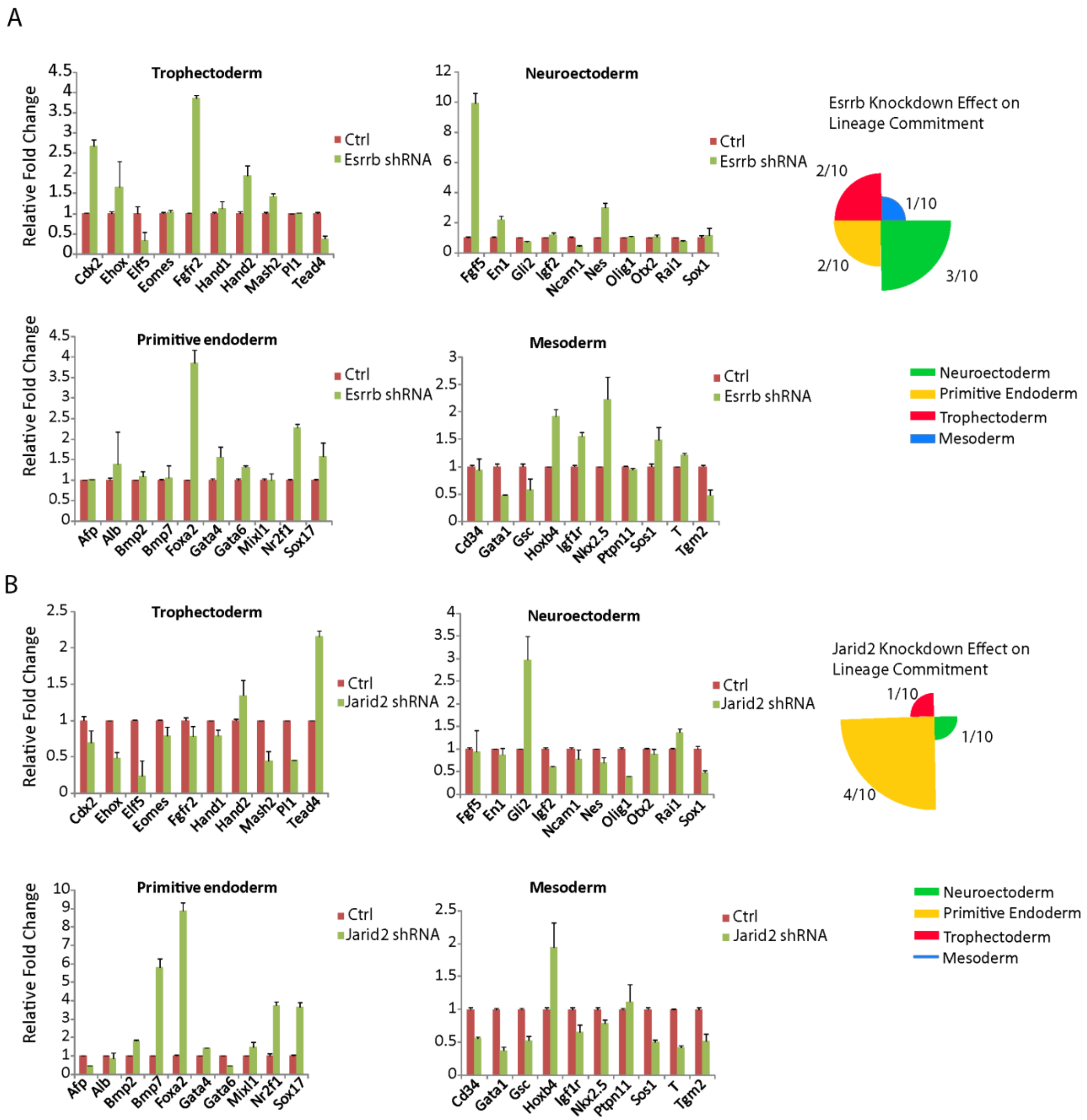

Figure S5. Expression measurements of lineage specification markers after knockdowns in mESCs

Plots show the relative fold-change for 40 lineage marker genes after knockdowns by Esrrb-shRNA (A) and Jarid2-shRNA (B). Pie charts display each slice with radius proportional to the number of lineage specific genes with more than two fold-changes as compared with the controls. All values were normalized by the expression of *Gapdh*.
