# Supplementary material for: Construction and Validation of a Regulatory Network for Pluripotency and Self-Renewal of Mouse Embryonic Stem Cells
Source: PLoS Comput Biol. 2014 Aug 14;10(8):e1003777. doi: 10.1371/journal.pcbi.1003777 (PMC4133156; doi:10.1371/journal.pcbi.1003777)
Supplement: Figure S6 — Ensemble and comparison of learned networks from single cell datasets. (PDF) [file pcbi.1003777.s006.pdf]

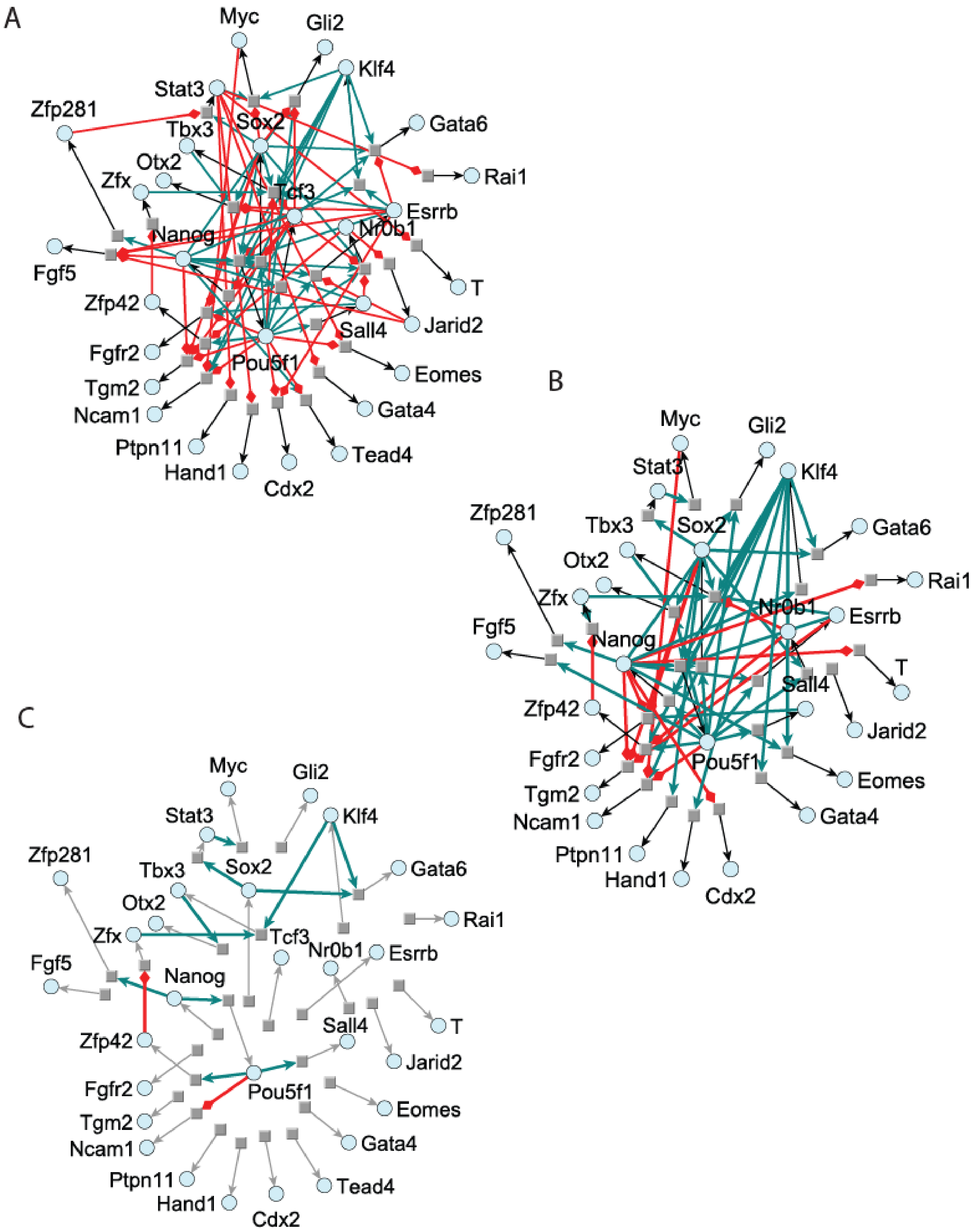

Figure S6. Ensemble and comparison of learned networks from single cell datasets

(A-B) Ensembles of learned networks. Learning was achieved using the (A) CCE single cell data and (B) Nanog-rescue single cell data. Light cyan nodes represent genes; gray squares represent learned regulatory logic transition functions. Links from upstream parent nodes appearing in more than 90% of equally well-fitted Boolean functions are colored in green (activation) and red (repression). Other links are not shown. (C) Overlap among four ensemble networks learned from the serum/LIF, 2i/LIF, CCE and Nanog-rescue single cell dataset, respectively. Links that are consistent across all four models are shown.
