# Supplementary material for: Construction and Validation of a Regulatory Network for Pluripotency and Self-Renewal of Mouse Embryonic Stem Cells
Source: PLoS Comput Biol. 2014 Aug 14;10(8):e1003777. doi: 10.1371/journal.pcbi.1003777 (PMC4133156; doi:10.1371/journal.pcbi.1003777)
Supplement: Figure S7 — Illustration of quantifying agreement between simulation and experiment results. (PDF) [file pcbi.1003777.s007.pdf]

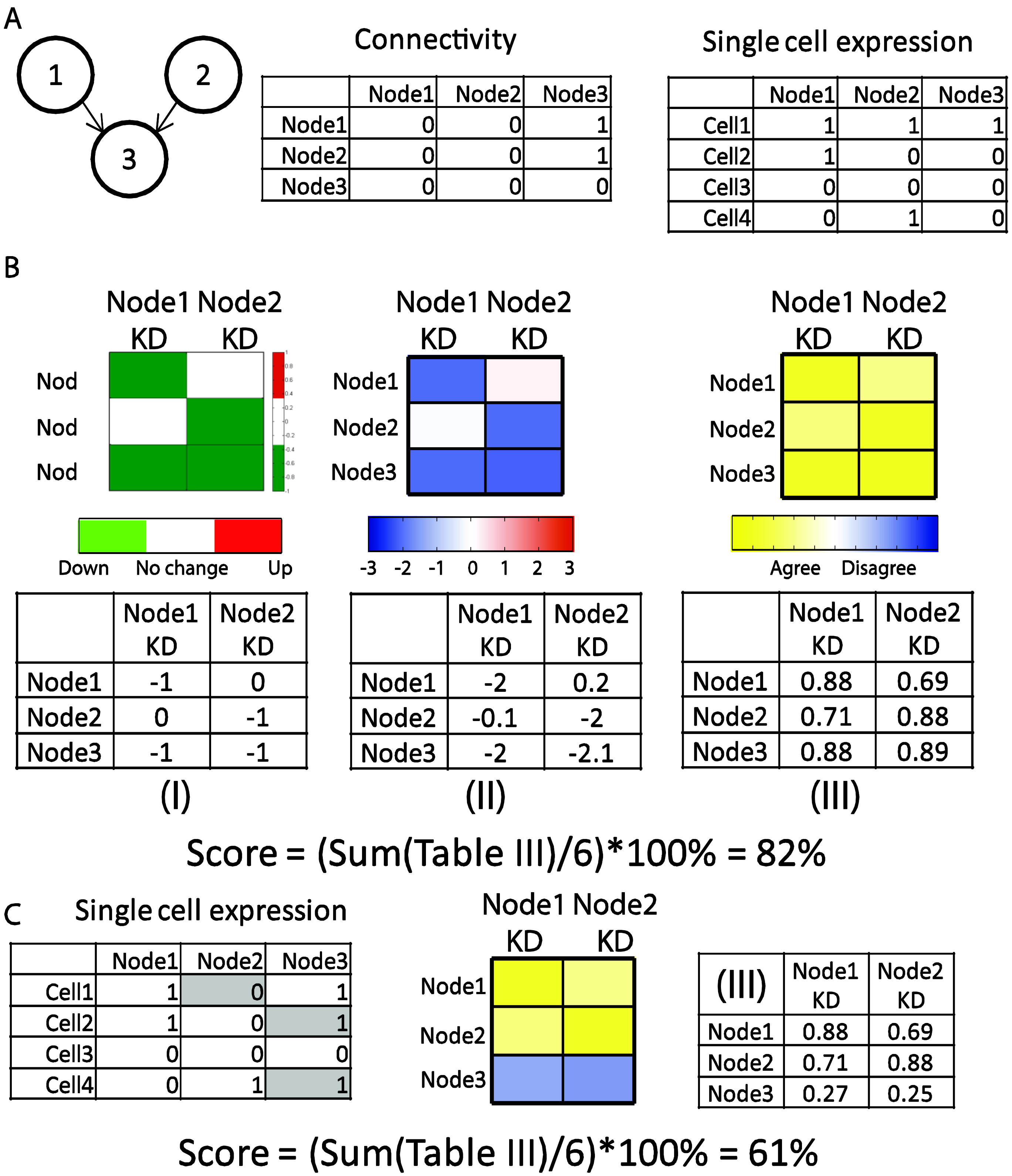

Figure S7 Illustration of quantifying agreement between simulation and experiment results

(A) A pseudo directed network with three nodes. The connectivity table represents the adjacency matrix underlying the network and the single cell expression table represents pseudo binary expression level of the three nodes in four individual cells. (B) Heatmaps (upper) were generated the same way as in Figure 2-14, with the corresponding tables (lower) representing the underlying values. A score is calculated the same way as the relative accuracy score of the y-axis in Figure 2-18. (C) Three entries (grey) of the single cell expression data were flipped and used as input for learning the regulatory logic. The heatmap and the corresponding table were generated the same way as in III of B.
