# Supplementary material for: Construction and Validation of a Regulatory Network for Pluripotency and Self-Renewal of Mouse Embryonic Stem Cells
Source: PLoS Comput Biol. 2014 Aug 14;10(8):e1003777. doi: 10.1371/journal.pcbi.1003777 (PMC4133156; doi:10.1371/journal.pcbi.1003777)
Supplement: Table S1 — Literature evidence and processing methods for extraction of interactions. (PDF) [file pcbi.1003777.s008.pdf]

Gene Name	LOF_EVID_ PMID	LOF_Criteria	GOF_EVID _PMID	GOF_ Criteria	ChIP_EVID
**Esrrb**	16767105	FC>=2&abs(exp)>300& #P_calls>=3(literature)	NA	NA	18555785,unpublished
	19136965	Bscore>0&fc>1.5			
**Klf4**	18264089	FC>1.5& q-value<0.05(literature)	19796622	FC=1.5& FDR<=0.05	18264089, 18358810, 19030024, 18555785
**Myc**	NA	NA	19796622	FC=1.5& FDR<=0.05	18358816, 19079543, 18555785, 19030024
**Nanog**	16518401	FDR<0.001(literature)&fc>=2	19796622	FC=1.5& FDR<=0.05	16518401, 18347094, 18358816, 18692474, 18700969, 18555785
	16767105	FC>=2&abs(exp)>300& #P_calls>=3(literature)			
**Nr0b1**	19530134	FDR<11.9%_24hr_kd& FDR<13.8%_48hr_kd (literature)	19796622	FC=1.5& FDR<=0.05	18358816
**Pou5f1**	16518401	FDR<0.001(literature)&FC>=2	19796622	FC=1.5& FDR<=0.05	16518401, 18347094, 18358816, 18692474, 18700969, 18555785
	16767105	FC>=2&abs(exp)>300& #P_calls>=3(literature)			
**Sall4**	19350679	detection_call=p&FC>=2	19796622	FC=1.5& FDR<=0.05	18804426
**Sox2**	16767105	FC>=2&abs(exp)>300& #P_calls>=3(literature)	19796622	FC=1.5& FDR<=0.05	18358816, 18692474, 19030024, 18555785
	17515932	FDR <0.05&FC>1.5(literature)			
**Stat3**	NA	NA	19796622	FC=1.5& FDR<=0.11	19079543, 18555785
			19544440	literature	
**Tbx3**	16767105	FC>=2&abs(exp)>300& #P_calls>=3(literature)	NA	NA	20139965
	20139965	FDR<=0.05			
**Tcf3**	20139965	FDR<=0.05&FC>1.3	19796622	FC=1.5& FDR<=0.05	18347094, 18692474, 18467660
**Zfp281**	18757296	FDR<=0.05(literature)	NA	NA	18358816
	wanglab_ unpublished	FDR<=0.05&FC>=2			
**Zfp42**	19618472	FC>=2(literature)	NA	NA	18358816
**Zfx**	17448993	Bscore>0&FC>1.5	NA	NA	18555785
**Jarid2**	20075857	FC>=2	NA	NA	20064375, 20075857

Table S1. Literature evidence and processing methods for extraction of interactions
