# Supplementary material for: Construction and Validation of a Regulatory Network for Pluripotency and Self-Renewal of Mouse Embryonic Stem Cells
Source: PLoS Comput Biol. 2014 Aug 14;10(8):e1003777. doi: 10.1371/journal.pcbi.1003777 (PMC4133156; doi:10.1371/journal.pcbi.1003777)
Supplement: Table S2 — References for modifications of interactions from low-throughput studies. (PDF) [file pcbi.1003777.s009.pdf]

Initial Interaction	Updated Interaction	Reference
**NA**	Esrrb -> Oct4/Pou5f1	18957414
**Nanog ---| Tcf3**	Nanog -> Tcf3	18483421
**Oct4/Pou5f1 ---| Sall4**	Oct4/Pou5f1 -> Sall4	20505821
**Oct4/Pou5f1 ---| Tcf3**	Oct4/Pou5f1 -> Tcf3	18483421
**NA**	Sall4->Oct4/Pou5f1	16980957
**NA**	Zfp281 -> Nanog	18757296
**NA**	Zfp281 -> Zfp282	18757296
**Sox2 ---| Oct4/Pou5f1**	Sox2 -> Oct4/Pou5f1	15988017

'—|' represents repression and '->' represents activation.

Table S2. References for modifications of interactions from low-throughput studies
