# Supplementary material for: Construction and Validation of a Regulatory Network for Pluripotency and Self-Renewal of Mouse Embryonic Stem Cells
Source: PLoS Comput Biol. 2014 Aug 14;10(8):e1003777. doi: 10.1371/journal.pcbi.1003777 (PMC4133156; doi:10.1371/journal.pcbi.1003777)
Supplement: Table S3 — Information about probes/genes used in the single cell Fluidigm experiments. (PDF) [file pcbi.1003777.s010.pdf]

Probe Number	Gene Symbol	Specific Markers	Assay ID	Amplicon Length	Ref Seq
**1**	Bmp4	Pluripotency	Mm00432087m1	61	NM_007554.2
**2**	Myc	Pluripotency	Mm00487803_m1*	83	NM_010849.4
**3**	Ctnnb1	Pluripotency	Mm00483033_m1	75	NM_007614.2
**4**	Dppa4	Pluripotency	Mm00835624_g1	100	NM_028610.2
**5**	Esrrb	Pluripotency	Mm00442411_m1*	55	2 RefSeqs
**6**	Fbxo15	Pluripotency	Mm01265526_m1*	72	NM_015798.3
**7**	Fgf4	Pluripotency	Mm00438917_m1*	109	NM_010202.5
**8**	Jarid2	Pluripotency	Mm00445560_m1	103	NM_021878.2
**9**	Kdm4c	Pluripotency	Mm00460578_m1*	61	NM_144787.1
**10**	Klf4	Pluripotency	Mm00516104_m1*	77	NM_010637.3
**11**	Lif	Pluripotency	Mm00434761_m1	64	NM_008501.2
**12**	Lin28	Pluripotency	Mm00524077_m1*	79	NM_145833.1
**13**	Mbd3	Pluripotency	Mm00488961_m1*	69	NM_013595.2
**14**	Nacc1	Pluripotency	Mm01251609_g1*	146	NM_025788.2
**15**	Nanog	Pluripotency	Mm01617762_g1	82	NM_028016.2
**16**	Nr0b1	Pluripotency	Mm00431729_m1*	78	NM_007430.4
**17**	Nr5a2	Pluripotency	Mm00446088_m1*	104	2 RefSeqs
**18**	Pou5f1	Pluripotency	Mm00658129_gH	148	NM_013633.2
**19**	Prmt6	Pluripotency	Mm00619134_m1	83	NR_024139.1
**20**	Rest	Pluripotency	Mm00803268_m1	76	
**21**	Sall4	Pluripotency	Mm01240680_m1*	66	3 RefSeqs
**22**	Setdb1	Pluripotency	Mm00450791_m1*	76	
**23**	Smad1	Pluripotency	Mm00484721_m1*	63	NM_008539.3
**24**	Smarcc1	Pluripotency	Mm00486224_m1*	63	NM_009211.2
**25**	Socs3	Pluripotency	Mm01249143_g1	113	NM_007707.2
**26**	Sox2	Pluripotency	Mm00488369_s1*	68	NM_011443.3
**27**	Stat3	Pluripotency	Mm01219775_m1*	75	3 RefSeqs
**28**	Tbx3	Pluripotency	Mm00809779_s1	132	2 RefSeqs
**29**	Tcf3	Pluripotency	Mm01188714_m1*	66	2 RefSeqs
**30**	Tcfcp2l1	Pluripotency	Mm00470119_m1*	78	NM_023755.2
**31**	Tcl1	Pluripotency	Mm00493477_m1*	89	NM_009337.2
**32**	Tdgf1	Pluripotency	Mm00783944_g1	118	
**33**	Tert	Pluripotency	Mm00436931_m1	110	NM_009354.1
**34**	Trp53	Pluripotency	Mm01731287_m1*	133	2 RefSeqs
**35**	Wdr5	Pluripotency	Mm01332446_m1*	74	NM_080848.2
**36**	Zfp281	Pluripotency	Mm01296016_s1*	79	2 RefSeqs
**37**	Zfp42	Pluripotency	Mm01194090_g1	81	NM_009556.3
**38**	Zfp57	Pluripotency	Mm00456405_m1*	77	2 RefSeqs
**39**	Zfx	Pluripotency	Mm00494277_s1	75	2 RefSeqs
**40**	En1	Ectoderm	Mm00438709_m1*	90	NM_010133.2
**41**	Fgf5	Ectoderm	Mm00438919_m1	135	NM_010203.4
**42**	Gfap	Ectoderm	Mm00546086_m1	70	NM_010277.3
**43**	Gli2	Ectoderm	Mm01293117_m1	62	NM_001081125.1
**44**	Igf2	Ectoderm	Mm00439564_m1*	107	3 RefSeqs
**45**	Lhx3	Ectoderm	Mm01333633_m1*	94	NM_001039653.1
**46**	Nav1	Ectoderm	Mm00557911_m1*	98	NM_173437.2
**47**	Ncam1	Ectoderm	Mm00456815_m1	83	NM_010875.3
**48**	Nes	Ectoderm	Mm00450205_m1*	72	NM_016701.3
**49**	Nog	Ectoderm	Mm00476456_s1	125	NM_008711.2
**50**	Notch3	Ectoderm	Mm01345646_m1	83	NM_008716.2
**51**	Olig1	Ectoderm	Mm00497537_s1*	83	NM_016968.4
**52**	Otx2	Ectoderm	Mm00446859_m1*	74	NM_144841.3
**53**	Pax6	Ectoderm	Mm00443072_m1*	56	NM_013627.4
**54**	Pten	Ectoderm	Mm00477210_m1	90	NM_008960.2
**55**	Rai1	Ectoderm	Mm01163529_m1*	85	2 RefSeqs
**56**	Smo	Ectoderm	Mm01162710_m1*	58	NM_176996.4
**57**	Sox1	Ectoderm	Mm00486299_s1*	71	NM_009233.3
**58**	Sox10	Ectoderm	Mm01300162_m1*	77	NM_011437.1
**59**	Ppp3ca	Mesoderm	Mm01317678_m1*	92	NM_008913.4
**60**	Cd34	Mesoderm	Mm00519283_m1*	61	2 RefSeqs

**61**	Gata1	Mesoderm	Mm01352636_m1*	73	NM_008089.1
**62**	Gsc	Mesoderm	Mm00650681_g1*	122	NM_010351.1
**63**	Hoxb4	Mesoderm	Mm00657964_m1*	116	NM_010459.6
**64**	Igf1r	Mesoderm	Mm00802841_m1	69	NM_010513.2
**65**	Isl1	Mesoderm	Mm00627860_m1	99	NM_021459.4
**66**	Kdr	Mesoderm	Mm00440105_m1	74	NM_010612.2
**67**	Mef2a	Mesoderm	Mm01318991_m1*	64	NM_001033713.1
**68**	Myf5	Mesoderm	Mm004345125	71	NM_008656.5
**69**	Myh7b	Mesoderm	Mm01249956_g1	111	NM_001085378.1
**70**	Myod1	Mesoderm	Mm00440387_m1*	86	NM_010866.2
**71**	Myog	Mesoderm	Mm00446194_m1*	69	NM_031189.2
**72**	Nkx2-5	Mesoderm	Mm00657783_m1	117	NM_008700.2
**73**	Pdgfra	Mesoderm	Mm01211694_m1	72	2 RefSeqs
**74**	Ptpn11	Mesoderm	Mm00448434_m1*	80	2 RefSeqs
**75**	Sos1	Mesoderm	Mm00436731_m1*	56	NM_009231.2
**76**	T	Mesoderm	Mm00436877_m1	86	NM_009309.2
**77**	Tgm2	Mesoderm	Mm00436987_m1*	72	NM_009373.3
**78**	Trib3	Mesoderm	Mm00454879_m1*	79	NM_175093.2
**79**	Afp	Endoderm	Mm00431715_m1*	96	NM_007423.4
**80**	Alb	Endoderm	Mm00802090_m1*	89	NM_009654.3
**81**	Bmp7	Endoderm	Mm00432102_m1*	72	NM_007557.2
**82**	Foxa2	Endoderm	Mm01976556_s1*	73	NM_010446.2
**83**	Gata4	Endoderm	Mm00484689_m1*	84	NM_008092.3
**84**	Gata6	Endoderm	Mm00802636_m1*	120	NM_010258.3
**85**	Mixl1	Endoderm	Mm00489085_m1*	74	NM_013729.3
**86**	Sox17	Endoderm	Mm00488363_m1*	86	NM_011441.4
**87**	Cdx2	Trophectoderm	Mm00432449_m1	88	NM_007673.3
**88**	Elf5	Trophectoderm	Mm00468732_m1*	105	2 RefSeqs
**89**	Eomes	Trophectoderm	Mm01351985_m1*	58	NM_010136.2
**90**	Fgfr2	Trophectoderm	Mm00438941_m1	87	2 RefSeqs
**91**	Hand1	Trophectoderm	Mm00433931_m1*	91	NM_008213.2
**92**	Pgf	Trophectoderm	Mm00435613_m1*	75	NM_008827.2
**93**	Tead4	Trophectoderm	Mm01189836_m1*	75	2 RefSeqs
**94**	Gapdh	Loading Control	Mm99999915_g1*	107	NM_008084.2
**95**	Actb	Loading Control	Mm02619580_g1	143	NM_007393.3
**96**	H_2O	Negative Control			

Table S3. Information about probes/genes used in the single cell Fluidigm experiments
