# Supplementary material for: Construction and Validation of a Regulatory Network for Pluripotency and Self-Renewal of Mouse Embryonic Stem Cells
Source: PLoS Comput Biol. 2014 Aug 14;10(8):e1003777. doi: 10.1371/journal.pcbi.1003777 (PMC4133156; doi:10.1371/journal.pcbi.1003777)
Supplement: Table S4 — Boolean function distribution and degeneracy counts. (PDF) [file pcbi.1003777.s011.pdf]

Gene Name	# of Boolean functions (serum-data)	# of Boolean functions (2i-data)	overlap Boolean functions	Reassign (serum-data)	Reassign (2i-data)	include-self-loop (serum-data)	include-self-loop (2i-data)
**Esrrb**	5	8	3				
**Klf4**	1	2	0				
**Myc**	1	1	1				
**Nanog**	5	4	4				
**Nr0b1**	32	2	2				
**Pou5f1**	383	742	348				
**Sall4**	2	2	2				
**Sox2**	5	12	5				
**Stat3**	1	2	1				
**Tbx3**	44	9	0				
**Tcf3**	2	2	1				
**Zfp281**	1	1	1				
**Zfp42**	94	75	75				
**Zfx**	1	1	1			YES	YES
**Jarid2**	1	1	0	YES			
**Cdx2**	2	1	1				
**Eomes**	197	66	66				
**Fgf5**	54	17	0				
**Fgfr2**	197	138	113				
**Gata4**	1	1	0	YES	YES		
**Gata6**	1	1	1				
**Hand1**	1	1	0	YES	YES		
**Otx2**	47	23771	0				
**T**	1	1	1				
**Tead4**	2	2	2				
**Gli2**	5	10	0				
**Ncam1**	18	5	5				
**Ptpn11**	1	1	0	YES			
**Rai1**	1	1	1				
**Tgm2**	197	28	24				

Table S4. Boolean function statistics

The table lists the number of total learned Boolean functions, auto-regulation and reassignments of links.
