# Supplementary material for: Construction and Validation of a Regulatory Network for Pluripotency and Self-Renewal of Mouse Embryonic Stem Cells
Source: PLoS Comput Biol. 2014 Aug 14;10(8):e1003777. doi: 10.1371/journal.pcbi.1003777 (PMC4133156; doi:10.1371/journal.pcbi.1003777)
Supplement: Table S5 — Comparing expression levels for genes in serum/LIF vs 2i/LIF. (PDF) [file pcbi.1003777.s012.pdf]

GeneName	Serum/LIF	2i/LIF	Serum - 2i
Esrrb	-5.80	-6.29	0.49
Klf4	-7.39	-4.61	-2.77
Myc	-13.32	-15.97	2.65
Nanog	-4.37	-3.44	-0.93
Nr0b1	-13.00	-14.11	1.12
Pou5f1	-2.22	-4.14	1.92
Sall4	-8.63	-8.68	0.05
Sox2	-5.79	-11.31	5.52
Stat3	-6.45	-6.49	0.04
Tbx3	-11.91	-9.85	-2.07
Tcf3	-8.22	-9.93	1.71
Zfp281	-4.30	-5.53	1.23
Zfp42	-5.29	-4.40	-0.89
Zfx	-9.72	-11.40	1.68
Jarid2	-3.22	-6.58	3.36
Cdx2	-15.93	-18.93	3.00
Eomes	-12.34	-15.02	2.69
Fgf5	-13.75	2.03	-15.79
Fgfr2	-16.07	-18.61	2.54
Gata4	-15.30	-18.93	3.63
Gata6	-15.71	-18.83	3.12
Hand1	-16.19	-18.93	2.74
Otx2	-12.00	4.46	-16.46
T	-16.14	-18.93	2.79
Tead4	-14.00	-14.99	0.99
Gli2	-13.72	-13.23	-0.50
Ncam1	-16.12	-18.93	2.81
Ptpn11	-12.95	-14.39	1.44
Rai1	-13.53	-17.51	3.98
Tgm2	-14.07	-15.45	1.38

Table S5. 30 gene average (-delta Ct) expression in serum/LIF, 2i/LIF and difference between the two.
