# Supplementary material for: Construction and Validation of a Regulatory Network for Pluripotency and Self-Renewal of Mouse Embryonic Stem Cells
Source: PLoS Comput Biol. 2014 Aug 14;10(8):e1003777. doi: 10.1371/journal.pcbi.1003777 (PMC4133156; doi:10.1371/journal.pcbi.1003777)
Supplement: Table S6 — Out-degree centrality measures in terms of critical links of nodes in the ensemble, representative networks of mESCs in serum/LIF and 2i/LIF. (PDF) [file pcbi.1003777.s013.pdf]

Gene Name	Out-degree (serum)	Out-degree (2i)
Esrrb	4	6
Klf4	4	3
Myc	0	0
Nanog	6	6
Nr0b1	2	2
Pou5f1	15	13
Sall4	1	1
Sox2	6	8
Stat3	1	1
Tbx3	1	1
Tcf3	5	7
Zfp281	1	0
Zfp42	1	1
Zfx	2	2
Jarid2	1	0

Table S6. Out-degree centrality measures in terms of critical links of nodes in the ensemble, representative networks of mESCs in serum/LIF and 2i/LIF
