# Supplementary material for: Construction and Validation of a Regulatory Network for Pluripotency and Self-Renewal of Mouse Embryonic Stem Cells
Source: PLoS Comput Biol. 2014 Aug 14;10(8):e1003777. doi: 10.1371/journal.pcbi.1003777 (PMC4133156; doi:10.1371/journal.pcbi.1003777)
Supplement: Table S7 — Relationship between protein pairs connected by ‘AND’ gate and literature evidence. (PDF) [file pcbi.1003777.s014.pdf]

(A)

Interacting Protein A	Interacting Protein B	'AND' Gate
Esrrb	Pou5f1	1
Esrrb	Sall4	1
Esrrb	Nr0b1	1
Esrrb	Nanog	1
Esrrb	Sox2	1
Klf4	Pou5f1	1
Klf4	Sox2	1
Nanog	Pou5f1	1
Nanog	Zfp281	0
Nanog	Nr0b1	1
Nanog	Sall4	1
Nanog	Tbx3	0
Nanog	Sox2	1
Nr0b1	Pou5f1	1
Nr0b1	Sall4	0
Pou5f1	Sall4	1
Pou5f1	Sox2	1
Pou5f1	Zfp42	0
Sall4	Zfp281	0
Sall4	Sox2	1

(B)

Rank	AND Connected ProteinA	AND Connected ProteinB	Frequency of Occurrence	Literature Support?	Reference<br>Large-scale IP-MS	Small-scale co-IP
**1**	Esrrb	Sox2	4.83E-02	Yes	22334693;	
**2**	Nanog	Sox2	4.53E-02	Yes	21589869;22334693;	
**3**	Esrrb	Nanog	3.91E-02	Yes	18957414;	17093407
**4**	Pou5f1	Sox2	2.76E-02	Yes	20362541;15863505;16978048;<br>17324942;22334693	22334693
**5**	Klf4	Sox2	2.38E-02	Yes	22334693;	19816951
**6**	Esrrb	Pou5f1	2.38E-02	Yes	20362541;	18662995
**7**	Esrrb	Tcf3	2.30E-02	No		
**8**	Sox2	Tcf3	2.23E-02	No		
**9**	Klf4	Nanog	2.07E-02	No		
**10**	Klf4	Sall4	2.07E-02	No		

Table S7. Relationship between protein pairs connected by 'AND' gate and literature evidence (A) Summary of the known protein-protein interactions extracted from ESCAPE and whether supported by the 'AND' gate in the learned transition functions (indicated in the 3rd column by '1' for being connected by 'AND' and '0' otherwise). (B) Summary of the top 10 most frequent 'AND' connected pairs in all learned Boolean functions and with indication of supporting by known protein-protein interactions.
