# Supplementary material for: Construction and Validation of a Regulatory Network for Pluripotency and Self-Renewal of Mouse Embryonic Stem Cells
Source: PLoS Comput Biol. 2014 Aug 14;10(8):e1003777. doi: 10.1371/journal.pcbi.1003777 (PMC4133156; doi:10.1371/journal.pcbi.1003777)
Supplement: Table S8 — Comparison values of computational and experimental knockdowns used in Figure 5D. (PDF) [file pcbi.1003777.s015.pdf]

Gene	Ei	Ni	Oi	EOi	ENi	ONi	NEOi
**Esrrb**	0.25	0.34	0.24	0.24	0.21	0.21	0.21
**Klf4**	0.37	0.29	0.39	0.41	0.39	0.38	0.60
**Myc**	0.47	0.27	0.31	0.38	0.56	0.35	0.57
**Nanog**	0.31	0.17	0.31	0.32	0.24	0.24	0.33
**Nr0b1**	0.48	0.30	0.51	0.46	0.48	0.42	0.36
**Pou5f1**	0.28	0.33	0.14	0.17	0.29	0.17	0.20
**Sall4**	0.33	0.31	0.50	0.50	0.37	0.44	0.59
**Sox2**	0.27	0.32	0.41	0.31	0.27	0.30	0.44
**Stat3**	0.36	0.45	0.39	0.47	0.52	0.45	0.58
**Tbx3**	0.40	0.32	0.33	0.36	0.51	0.31	0.33
**Tcf3**	0.37	0.34	0.70	0.66	0.44	0.61	0.80
**Zfp281**	0.34	0.61	0.61	0.60	0.68	0.59	0.77
**Zfp42**	0.36	0.32	0.51	0.47	0.41	0.51	0.36
**Zfx**	0.37	0.43	0.36	0.38	0.49	0.45	0.60
**Jarid2**	0.28	0.31	0.32	0.39	0.28	0.46	0.27
**Cdx2**	0.72	0.58	0.02	0.03	0.89	0.08	0.09
**Eomes**	0.28	0.45	0.50	0.45	0.45	0.47	0.59
**Fgf5**	0.74	0.45	0.57	0.55	0.56	0.30	0.11
**Fgfr2**	0.34	0.57	0.24	0.36	0.23	0.34	0.15
**Gata4**	0.34	0.46	0.32	0.28	0.53	0.28	0.16
**Gata6**	0.32	0.41	0.43	0.34	0.46	0.40	0.67
**Hand1**	0.29	0.67	0.04	0.15	0.65	0.04	0.06
**Otx2**	0.42	0.39	0.19	0.14	0.37	0.17	0.21
**T**	0.48	0.35	0.27	0.48	0.35	0.41	0.13
**Tead4**	0.28	0.39	0.36	0.39	0.44	0.31	0.45
**Gli2**	0.42	0.45	0.32	0.38	0.48	0.53	0.40
**Ncam1**	0.32	0.31	0.44	0.46	0.29	0.50	0.29
**Ptpn11**	0.36	0.42	0.30	0.26	0.54	0.31	0.14
**Rai1**	0.41	0.36	0.09	0.12	0.25	0.19	0.07
**Tgm2**	0.46	0.38	0.51	0.67	0.48	0.63	0.47

Table S8. Comparison values of computational and experimental knockdowns used in Fig. 4D.
