# Supplementary material for: Construction and Validation of a Regulatory Network for Pluripotency and Self-Renewal of Mouse Embryonic Stem Cells
Source: PLoS Comput Biol. 2014 Aug 14;10(8):e1003777. doi: 10.1371/journal.pcbi.1003777 (PMC4133156; doi:10.1371/journal.pcbi.1003777)
Supplement: Table S9 — Primers used for RT-PCR analysis in mESCs. (PDF) [file pcbi.1003777.s016.pdf]

Gene	Accesion Number	5' Primer	3'Primer
α-fetoprotein	NM_007423.4	GCCAAAGTGGAGTGGAAAGA	GCAGGTTCTGGAAACTGGAA
Albumin	NM_009654.3	TTCCAAACCTCCGTGAAAAC	GCTGGGGTTGTCATCTTTGT
Actin	NM_007393.3	ACCAACTGGGACGACATGGAGAAG	TACGACCAGAGGCATACAGGGACA
Ascl2 (Mash2)	NM_008554.3	AGATGACCTCTGTCCCTCACCC	CAACAGCAGGGTTCCCACAC
Bmp2	NM_007553.2	CGTCCTCAGCGAATTTGAGT	GCCTGCGGTACAGATCTAGC
Bmp7	NM_007557.2	ATTTCAGCCTGGACAACGAG	TTATGCTTTCCCTGGAGGTG
Cd34	NM_001111059.1	CCCTGGCAGATTCCTTTCAGTC	CCCAATCCTCTCATCTCTGGAAAG
Cdx2	NM_007673.3	ACCTTCTGGACAAGGACGTG	GCGGAGGACTGACAAAGTTC
Coup TF1	MMCOUP	ACCCAATCCTGCTCCCTGACTC	CTCATGGTCCTCTTCCAAATCG
Ehox	NM_021300.2	AACAGCAACCAAGAGGACCAGGAC	CTCTGGCTTCACTCACACCTATCC
Elf5	NM_010125.3	TCTGCTGCGACCAGTACAAG	GCTGCCTCAATGAACTCCTC
En1	NM_010133.2	GATTTCGGTTGCAAAAAGGA	CAGAGAGTGAACGGGGTCTC
Eomes	NM_001164789.1	TGCAAGAGAAAGCGCCTGTCTC	CAATCCAGCACCTTGAACGACC
Esrrb	NM_011934.4	CTAGTTGCGGCTCCTTCATC	TGGCGTTAAGCATGTACTCG
Fgf5	NM_010203.4	GCGACGTTTTCTTCGTCTTC	CTGTATGGACCCACAGGGAGTAAC
Fgfr2	NM_010207.2	CACCGAGAAGATGGAGAAGC	GCGATGCTCCTGCTTAAACT
Foxa2	NM_010446.2	GGCCCAGTCACGAACAAAGC	CCCAAAGTCTCCACTCAGCCTC
Gapdh	NM_001001303	ACTCCACTCACGGCAAATTC	TCTCCATGGTGGTGAAGACA
Gata1	NM_008089.1	GCACTCTACCCTGCCTCAAC	GCTCTTCCCTTCCTGGTCTT
Gata4	NM_008092.3	TTCCTGCTCGGACTTGGGAC	TTCCCAGGCAGGTGGAGAATAAG
Gata6	NM_010258.3	ACAGCCCACTTCTGTGTTCCC	GTGGGTTGGTCACGTGGTACAG
Gli2	NM_001081125.1	CAACGCCTACTCTCCCAGAC	GAGCCTTGATGTACTGTACCAC
Goosecoid	NM_010351.1	AAACGCCGAGAAGTGGAACAAG	AAGGCAGGGTGTGTGCAAGTAG
Hand1	NM_008213.2	AGAGGAGACGCACAGAGAGC	AGCACGTCCATCAAGTAGGC
Hand2	NM_010402.4	CGAGGAGAACCCCTACTTCC	GTACTCGGGGCTGTAGGACA
Hoxb4	NM_010459.7	CAGGTCCTGGAGTTGGAGAA	GTTGGGCAACTTGTGGTCTT
Igf1r	NM_010513.2	GAGAGCCTGGGAGACCTCTT	TAACAGAGGTCGGCGTTCTT
Igf2	NM_010514.3	GAGTTCAGAGAGGCCAAACG	CACTGATGGTTGCTGGACAT
Jarid2	NM_001205043.1	ACAATGCTTCATCTTCGTGCC	GCTCTTTCTCCCGTGCTGAC
Klf4	NM_010637.3	GTGCAGCTTGCAGCAGTAAC	AGCGAGTTGGAAAGGATAAAGTC
Mixl1	NM_013729.3	CACGCAGTGCTTTCCAAAC	GAAGACGAGCTCCAGCAACT
Myc	NM_010849.4	ACTACGACTCCGTACAGCCCTAT	TTTCTTCCAGATATCCTCACTGG
Nanog	XM_132755,XP_132755	CCAGTCCCAAACAAAAGCTC	ATCTGCTGGAGGCTGAGGTA
Ncam1	NM_001081445.1	GACAGAACCCGAAAAGGGC	GTTGGGGACCGTCTTGACTT
Nestin	NM_016701.3	CTCGGGAGAGTCGCTTAGAG	GCCTTCCAATCTCTGTTCCA
Nkx2.5	NM_008700.2	AAGCAACAGCGGTACCTGTC	GCTGTCGCTTGCACTTGTAG
Nr0b1	NM_007430.4	AAGTACTTGCCCTGCTTCCA	TCGAAGTGCAGGTGATCTTG
Oct4/Pou5f1	NM_013633	GGATGGCATACTGTGGACCT	GTTGGTTCCACCTTCTCCAA
Olig1	NM_016968.4	GCTGCGCGAAGTTATCCTAC	ACCCAGCAGCAGGATGTAGT
Otx2	NM_144841.3	TCTGACCCCTTGTCCACTTC	GAAGTTGAGCCAGCATAGCC

Pl1	NM_008864	GGGAGAATGTGTCCTCCAAA	ATCTGCGGCCAAGATAAATG
Ptpn11	NM_011202.3	AGAGGGAAGAGCAAATGTGTCA	CTGTGTTTCCTTGTCCGACCT
Rai1	NM_009021.2	GACCATCTCGTGCTCCTATAAAG	TGTTTGGGGCACTTCAAAGTAAA
Sall4	NM_175303.3	TCACCACGAAAGGCAACCTGAAGG	CATTCAGGACGCTGGTGTACTGGTT
Sos1	NM_009231.2	TGCCAAGTCAGCTGAAGAGA	CTCCTCGGAGTCAGGTTCTG
Sox1	NM_009233.3	ATGCACCGCTACGACATGGG	GCTCCGACTTGACCAGAGATCC
Sox17	NM_011441.4	TAAAGGTGAAAGGCGAGGTG	CTTAGCTCTGCGTTGTGCAG
Sox2	NM_011443.3	CACAACTCGGAGATCAGCAA	CTCCGGGAAGCGTGTACTTA
Stat3	NM_213659.2	GGAGGAGCTGCAGCAGAAAGTGTC	GACCAGCAACCTGACTTTCGTGGT
T	NM_009309.2	CTCTAATGTCCTCCCTTGTTGCC	TGCAGATTGTCTTTGGCTACTTTG
Tbx3	NM_011535.2	CGTCTCAGGCCTAGAATCCA	GTGTTGTTGGAGGTGGAAGG
Tcf3	NM_001164147.1	GCCATGAACGCTTCAATGTCCAG	CCTTCTTCACCGTAACTGGGGATTTC
Tead4	NM_011567.2	CGTCTCGGCCACAGCCTTCC	GCAGGAGACTCAAAGCCTGGCA
Tgm2	NM_009373.3	GACAATGTGGAGGAGGGATCT	CTCTAGGCTGAGACGGTACAG
Zfp281	NM_001160251.1	CCCCAGAGTATGGTTATGTTCAA	GTAGAGGAGGATAACACGCACTG
Zfp42	NM_009556.3	GCGGTGTGTACTGTGGTGTC	GACAAGCATGTGCTTCCTCA
Zfx	NM_001044386.1	ACCGTCCGGTGCGTATAA	TTCTCATCAGCCAGAACACCT

Table S9 Primers used for RT-PCR analysis in mESCs
