# Supplementary material for: Construction and Validation of a Regulatory Network for Pluripotency and Self-Renewal of Mouse Embryonic Stem Cells
Source: PLoS Comput Biol. 2014 Aug 14;10(8):e1003777. doi: 10.1371/journal.pcbi.1003777 (PMC4133156; doi:10.1371/journal.pcbi.1003777)
Supplement: Figure S2 — Characterization of gene expression of mESCs in serum/LIF and 2i/LIF at the single cell resolution. (PDF) [file pcbi.1003777.s002.pdf]

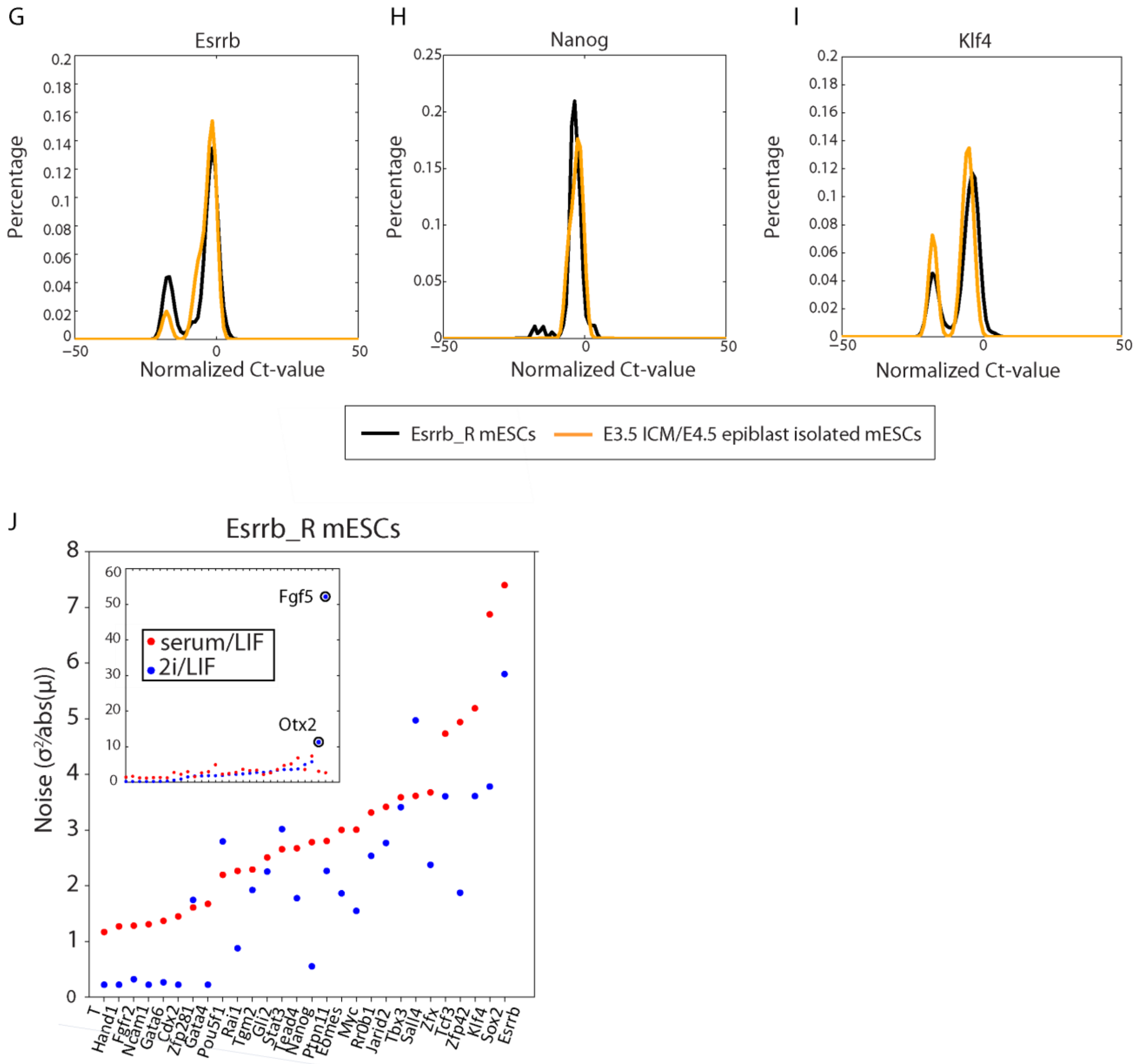

Figure S2. Characterization of gene expression of mESCs in serum/LIF and 2i/LIF at the single cell resolution

(A-B) Smoothened histograms of gene expression Ct-values from RT-PCR data collected from single mESCs cultured in serum/LIF (A) and 2i/LIF (B). Ct-values were normalized using the housekeeping gene *Gapdh* (the x-axis represents -ΔCt values and the y-axis represents the percentage of total cells). (C-D) Plots of normalized Ct-values partitioned into two clusters. (E-F) Hierarchical clustering of binarized gene expression values of 30 genes in 96 single mESCs in serum/LIF (E) and 2i/LIF (F). The x-axis represents single cells and the y-axis represents genes. Gray color denotes 'ON' or 'high' and black color denotes 'OFF' or 'low'. Orange bar indicates pluripotency and self-renewal genes and purple bar indicates lineage specific genes. (G-I) Comparison of the distribution of the expression of *Esrrb* (G), *Nanog* (H) and *Klf4* (I) between the Esrrb-rescue (Esrrb_R) mESCs (black line) and E3.5 ICM/E4.5 epiblast isolated mESCs (orange line). Profiles of gene expression at the single cell resolution in 14 ICM/epiblast isolated mESCs were obtained from Tang et al. [1]. The gene expression profile of Esrrb_R cell line was performed using Fluidigm platform-based single cell gene expression profiling. (J) Noise level of expression of the network genes in mESCs under serum/LIF and 2i/LIF media, based on single cell data of Esrrb_R mESCs. Inset in (J) include the noise levels of two outliers *Fgf5* and *Otx2* in the profiles of Esrrb_R.
