# Supplementary material for: Construction and Validation of a Regulatory Network for Pluripotency and Self-Renewal of Mouse Embryonic Stem Cells
Source: PLoS Comput Biol. 2014 Aug 14;10(8):e1003777. doi: 10.1371/journal.pcbi.1003777 (PMC4133156; doi:10.1371/journal.pcbi.1003777)
Supplement: Text S1 — Supporting information text including details about learning and optimization of Boolean transition functions, analysis of Oct4/Pou5f1 binding sites within gene promoter regions, comparison of distribution of Esrrb expression in Esrrb-rescue mESCs and other single mESCs, dynamical simulations and comparison between in silico and experimental knockdowns, quantifying agreement between experimental results and simulations results of networks learned from randomized single cell data/network topology, defining large sets of lineage-specific signature genes, lineage commitment predictions, and co-immuno-precipitation validation of Nanog-Sox2 interaction in ESCs. (PDF) [file pcbi.1003777.s017.pdf]

Supplemental Experimental Procedures

Learning and optimization of Boolean transition functions

A Boolean network $G(V,F)$ consists of a set of nodes $V=\{v_1,...v_n\}$ representing genes, whose activities can be either 0 (low) and 1 (high) and a list of Boolean functions $F=\{f_1, f_2,...f_n\}$, where $f_i(v_{i1},...,v_{ik})$ is defined with inputs $v_{i1},...v_{ik}$ from a specified subset of nodes assigned as inputs to node i. Let (I_j,O_j) be a pair of expression vectors of $\{v_1,..v_n\}$ in sample j, where we call I_j the Input from sample j and O_j the Output. For each node i we search for the Boolean function f_i with highest score $S(i,f_i)=\sum_{j=1}^{N}E_{i,j}$, where N is the total number of expression vectors, $E_{i,j}$ equals to 1 if $O_j(V_i)=f_i(I_j(V_{i1}),...,I_j(V_{ik}))$ is consistent with the status of node i in expression vector j and 0 otherwise. In other words, our goal is to identify the Boolean functions holding maximum consistency with the experimental observations. In order to identify these underlying Boolean functions with manageable search space and explicit interpretation, we attempted to enumerate all possible combinations of the logic operators AND, OR and NOT to connect each plausible upstream regulator to its target node. Inhibiting links in the network are interpreted as NOT-operations. Boolean functions are also constrained such that individual variables appear at most once in each function.

The following algorithm was used to search over possible Boolean functions:

Input: The initial network and binarized expression data from N single cells.

Loop: For each node i, explore all the possible combinations with the logic operators AND and OR connecting p plausible parents to node i based on the input network without considering self-loops. Inhibiting links in the input network are interpreted as NOT-gates. The Boolean function/s with the highest score are chosen across all possibilities from $1..p$ parents for each node.

Reassignment: If none of the combinations of plausible parents from the input network fulfills the criteria: $S(i,f_i)\geq 0.5\times \mathrm{N}$, where N is the number of single cell expression vectors, the algorithm exhaustively introduces single links from all the 15 pluripotency nodes. If still no single reassigned parent can explain the behavior of the downstream target by satisfying the $S(i,f_i)\geq 0.5\times \mathrm{N}$ criteria, we attempt all pairs of parents.

Analysis of Oct4/Pou5f1 binding sites within gene promoter regions

The position weight matrix of mouse Oct4/Pou5f1 was downloaded from JASPAR core database. 1000bp sequences upstream of transcription start site (TSS) of *Gata4*, *Hand1* and *Ptpn11* were downloaded from UCSC Genome Browser. The software ClusterDraw (Papatsenko, 2007) was applied to identify potential Oct4/Pou5f1-binding sites within the promoter region of *Gata4*, *Hand1* and *Ptpn11*.

Comparison of distribution of Esrrb expression in Esrrb-rescue mESCs and other single mESCs

Esrrb expression Ct-values were measured in single cells of 96 Esrrb-rescue clone mESCs cultured in serum/LIF by the Fluidigm microfluidic quantitative RT-PCR platform. In parallel, data were retrieved for *Esrrb* expression Ct-values in 14 mESCs measured from single cells by RT- PCR from a previously published study (Tang et al., 2010). Both datasets were normalized using the expression of the house-keeping gene *Gapdh*. Histograms for normalized Ct-values were smoothened using the Kernel smoothing algorithm in MATLAB, Statistics Toolbox. The consistency between the two histograms was assessed using the two-tailed Kolmogorov-Smirnov test (Young, 1977).

Defining large sets of lineage-specific signature genes

We generated lineage-specific signatures from two prior publications using the following criteria:

(1) Trophectoderm: the gene expression dataset (GSE11523) is from a study that reported trophectoderm-like state after depletion of Oct4/Pou5f1 in mESCs. Gene expression was profiled at six time-points. Genes were sorted according to average fold-change of expression upon differentiation related to time point 0. The top five percent of genes with an average fold-change of at least two and with a monotone increase in expression at each time point upon differentiation were considered as trophectoderm markers.

(2) Primitive endoderm: the same set of experiments and data processing as described for (1) were conduct after over-expression of *Gata6* in mESCs.

(3) Neuroectoderm: the gene expression dataset (GSE12982) is from a study that isolated Sox1-GFP positive cells from mESCs where Ezh1 and Ezh2 were knocked-down. Upon differentiation, Sox1-GFP positive cells were collected and profiled. Genes were sorted according to fold-change increase in expression comparing differentiated cells to mESCs. The top 10% genes with a monotonic increase and fold-change of at least 1.5 were considered as neuroectoderm markers.

(4) Mesendoderm: the same set of experiments and data processing as described for (3) were conduct after isolation of T-GFP positive cells (T stands for the gene *brachyury*).

Lineage commitment predictions

Effects of gene knockdowns (KD) on lineage commitment were quantified by enrichment analysis for KD effectors against lists of lineage-specific signature genes using the Fisher's exact test. For global knockdown effects, KD effectors were defined as genes in the set $(U_+ \cup D_-)\backslash(U_- \cup D_+)$; for direct knockdown effects, KD effectors were defined as genes in the set of $KD_-\backslash KD_+$, where U_+ (U_-) is the set of positive (negative) targets of up-regulated pluripotency regulators after the *in silico* knockdown; D_+ (D_-) is the set of positive (negative) targets of down-regulated pluripotency regulators after *in silico* knockdown; KD_- (KD_+) is the set of negative (positive) targets of the knocked-down factor(s); $A \cup B$ denotes the union of A and B; $A\backslash B$ denotes the set of all members in A but not B. All positive and negative targets were extracted from the loss-of-function table within the ESCAPE database.

Coimmunoprecipitation validation of Nanog-Sox2 interaction in ESCs

The *in-vivo* biotinylation version of Nanog was set in Nanog conditional knockout cell line, NGF/F. In these cells, the endogenous Nanog was replaced by flox flanked Nanog. Flag and biotin tagged Nanog was engineered into this cell line. GFP-Cre was used to remove flox flanked Nanog. BirAV5 was engineered into flbioNanog cells to biotinylate the biotin tag for affinity purification. Positive clones were picked and expanded, and expression of flbioNanog was confirmed by Western Blotting (WB) using streptavidin–HRP (GE Healthcare) and anti-Nanog antibody (Millipore). Standard procedures for ESC culture, the preparation of nuclear extract and streptavidin IP followed by WB are described as was done for previous studies (Ding et al.; Wang et al., 2006).

References

Ding, J., Xu, H., Faiola, F., Ma'ayan, A., and Wang, J. (2012) Oct4/Pou5f1 links multiple epigenetic pathways to the pluripotency network. Cell Res. 22, 155-167

Papatsenko, D. (2007). ClusterDraw web server: a tool to identify and visualize clusters of binding motifs for transcription factors. Bioinformatics 23, 1032-1034.

Tang, F., Barbacioru, C., Bao, S., Lee, C., Nordman, E., Wang, X., Lao, K., and Surani, M.A. (2010). Tracing the derivation of embryonic stem cells from the inner cell mass by single-cell RNA-Seq analysis. Cell Stem Cell 6, 468-478.

Wang, J., Rao, S., Chu, J., Shen, X., Levasseur, D.N., Theunissen, T.W., and Orkin, S.H. (2006). A protein interaction network for pluripotency of embryonic stem cells. Nature 444, 364-368.

Young, I.T. (1977). Proof without prejudice: use of the Kolmogorov-Smirnov test for the analysis of histograms from flow systems and other sources. J Histochem Cytochem 25, 935-941.
